# THP-1-derived macrophages render lung epithelial cells hypo-responsive to Legionella pneumophila – a systems biology study

**DOI:** 10.1038/s41598-017-12154-4

**Published:** 2017-09-20

**Authors:** Christine Schulz, Xin Lai, Wilhelm Bertrams, Anna Lena Jung, Alexandra Sittka-Stark, Christina Elena Herkt, Harshavadhan Janga, Katja Zscheppang, Christina Stielow, Leon Schulte, Stefan Hippenstiel, Julio Vera, Bernd Schmeck

**Affiliations:** 1 Institute for Lung Research, Universities of Giessen and Marburg Lung Center, Philipps-University Marburg, Member of the German Center for Lung Research (DZL), Marburg, Germany; 2 Laboratory of Systems Tumor Immunology, Department of Dermatology, Friedrich-Alexander-University of Erlangen-Nürnberg (FAU) and Universitätsklinikum Erlangen, Erlangen, Germany; 3 Institute for Lung Research/iLung, Research Group “RNA-Biology of Inflammation & Infection”, Philipps University, Marburg, Germany; 4 Department of Internal Medicine/Infectious Diseases and Respiratory Medicine, Charité – University Medicine Berlin, Berlin, Germany; 5 Department of Medicine, Pulmonary and Critical Care Medicine, University Medical Center Giessen and Marburg, Philipps-University Marburg, Member of the German Center for Lung Research (DZL), Marburg, Germany

## Abstract

Immune response in the lung has to protect the huge alveolar surface against pathogens while securing the delicate lung structure. Macrophages and alveolar epithelial cells constitute the first line of defense and together orchestrate the initial steps of host defense. In this study, we analysed the influence of macrophages on type II alveolar epithelial cells during *Legionella pneumophila*-infection by a systems biology approach combining experimental work and mathematical modelling. We found that *L*. *pneumophila*-infected THP-1-derived macrophages provoke a pro-inflammatory activation of neighboring lung epithelial cells, but in addition render them hypo-responsive to direct infection with the same pathogen. We generated a kinetic mathematical model of macrophage activation and identified a paracrine mechanism of macrophage-secreted IL-1β inducing a prolonged IRAK-1 degradation in lung epithelial cells. This intercellular crosstalk may help to avoid an overwhelming inflammatory response by preventing excessive local secretion of pro-inflammatory cytokines and thereby negatively regulating the recruitment of immune cells to the site of infection. This suggests an important but ambivalent immunomodulatory role of macrophages in lung infection.

## Introduction

Pneumonia has the highest mortality rate of all infectious diseases while exhibiting almost no improvements for the last decades^1–3^. Among young children, pneumonia still causes more deaths than malaria, AIDS, and measles together^4,5^.

Disease outcome critically depends on alveolar macrophages and pulmonary epithelial cells as the first line of defense^6,7^. Upon pathogen recognition, macrophage-derived cytokines such as TNF-α, IL-1α and IL-1β trigger the response to local infection by activating lung epithelial cells^8–10^. As a complementary line of defense, alveolar macrophages decelerate growth of internalized pathogens by apoptosis^11,12^.

However, pro-inflammatory mechanisms have to be carefully regulated, as organ function has to be maintained as well. Signalling cascades including interleukin-1 receptor-associated kinases (IRAK) and the transcription factor NF-κB are critical for balancing the initial innate immune response^13^. Accordingly, interruption of NF-κB signalling compromises host defense whereas enhanced NF-κB activity can lead to lung injury^10,14,15^.

It has been shown that cell-cell communication between macrophages and epithelial cells is an important early step in the immune response to respiratory tract infections^8,9^. However, not much is known about the detailed interaction of different cell types in pneumonia development. As one example, monocytes as well as epithelial cells were demonstrated to be activated through T-cell-derived IL-17 subsequently enabling neutrophil-mediated host defense^16^.

*Legionella pneumophila* (*L*. *pneumophila*) is an important pathogen of community-acquired pneumonia^17^. Its major target cells are alveolar macrophages^18^. In addition, *L*. *pneumophila* comes in close contact to and can infect alveolar epithelial cells^19,20^. Upon invasion through phagocytosis, within both host cells, *L*. *pneumophila* uses specialized secretion systems to create an intracellular legionella-containing vacuole, thereby evading lysosomal degradation and enabling intracellular replication^21,22^.

Communication between immune and non-immune cells during infection is a process tightly regulated at the temporal and spatial scale and controlled by paracrine and intracellular feedback loops, which are mediated by cytokine and chemokine signalling. Under these conditions, modelling and simulation under the systems biology paradigm can provide novel insights into key mechanisms and genes underlying cell-to-cell communication^23^.

In this study, we have analysed the crosstalk of human macrophages and lung epithelial cells during a sequential infection with *L*. *pneumophila*. Co-culture with infected macrophages reduced the pro-inflammatory reaction of epithelial cells upon subsequent *L*. *pneumophila* exposure. In a combined approach of experiments and mathematical modelling, we identified IL-1β-dependent IRAK-1 degradation as a central step in epithelial hypo-responsiveness.

## Results

### Infection of THP-1 cells with *L*. *pneumophila* induces cytokine release and paracrine activation of lung epithelial cells

**Figure 1:**
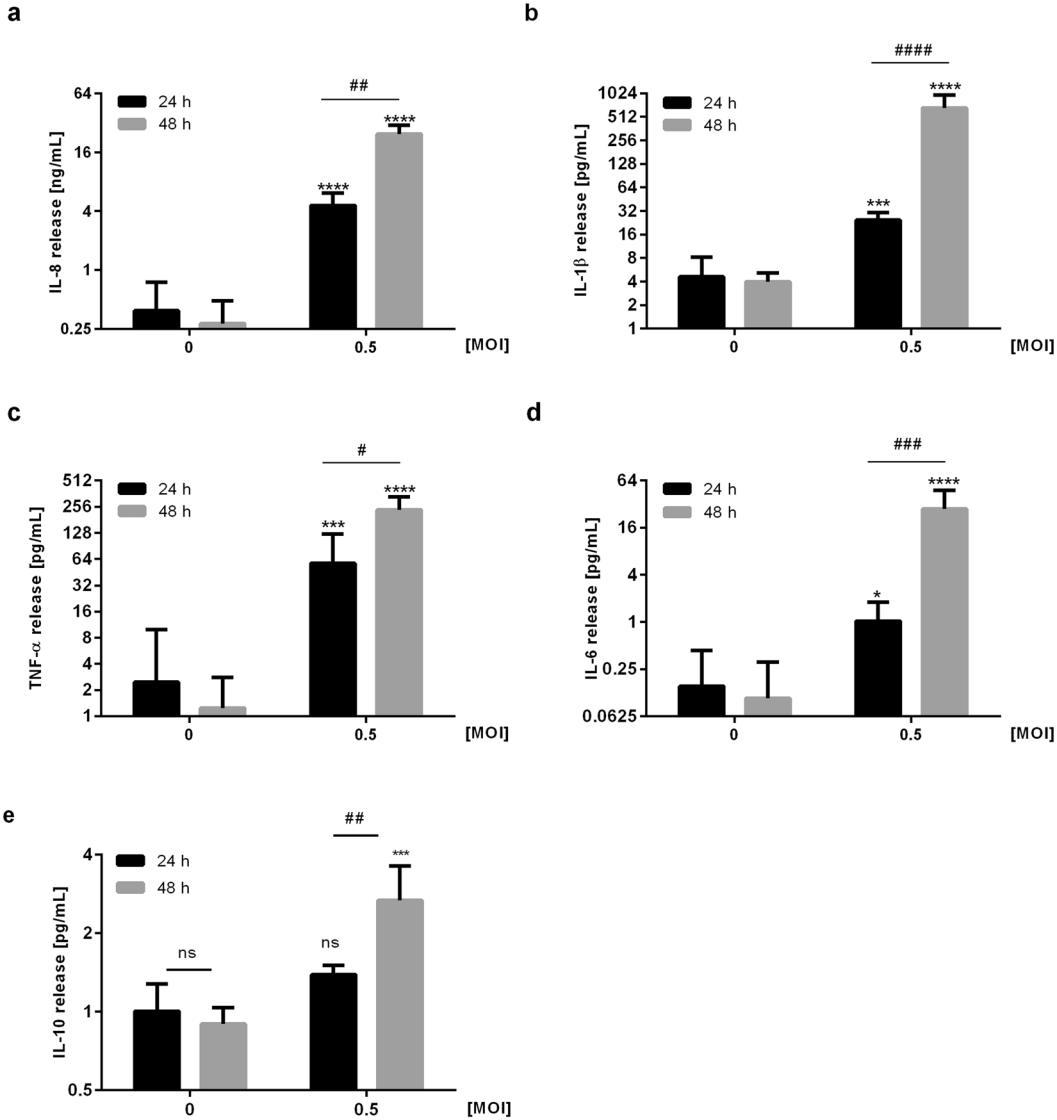
Infection of THP-1 cells with *L*. *pneumophila* induces pro-inflammatory cytokine release. THP-1 cells were stimulated with *L*. *pneumophila* (MOI 0.5) for 24 or 48 h, respectively. (**a**) IL-8 secretion was measured by ELISA, (**b**–**e**) secretion of IL-1β, TNF-α, IL-6 and IL-10 were measured using Multiplex Luminex Assay. Data are shown as mean ± SEM (n = 4). 2-Way ANOVA was performed with Sidak’s multiple comparison test as described: *Compared to corresponding control, ^#^compared to equally treated 24 h sample; ^#^or *p ≤ 0.05, ^##^p ≤ 0.01, ^###^or ***p ≤ 0.001, ****or ^####^p ≤ 0.0001.

To investigate the inflammatory response of macrophages to *L*. *pneumophila* infection, we analysed the secretion of pro-inflammatory cytokines in PMA-treated THP-1 cells upon infection with *L*. *pneumophila*. Cytokine secretion analyses demonstrated a significant induction of IL-8, IL-1β, TNF-α, IL-6 and IL-10 release 48 hours post infection (Fig. 1) To corroborate our findings, we measured the same cytokine panel after infection of human blood-derived macrophages with *L*. *pneumophila* (Supplementary Fig. S1) and observed an induction pattern similar to THP-1 cells.

Next, we examined the effect of cytokine treatment on the expression of pro-inflammatory genes in type II alveolar epithelial cells. Upon stimulation of A549 cells with IL-1β or TNF-α, respectively, *IL-8* expression significantly increased in a concentration-dependent manner showing its maximum twelve hours post stimulation (Supplementary Fig. S2a and b). However, the concentration of TNF-α needed for *IL-8* induction were approximately 40-fold higher than the cytokine amounts released by THP-1 cells in response to *L*. *pneumophila* infection (Supplementary Figs S2b and 1c). Therefore, TNF-α was considered less relevant in further experiments.

**Figure 2:**
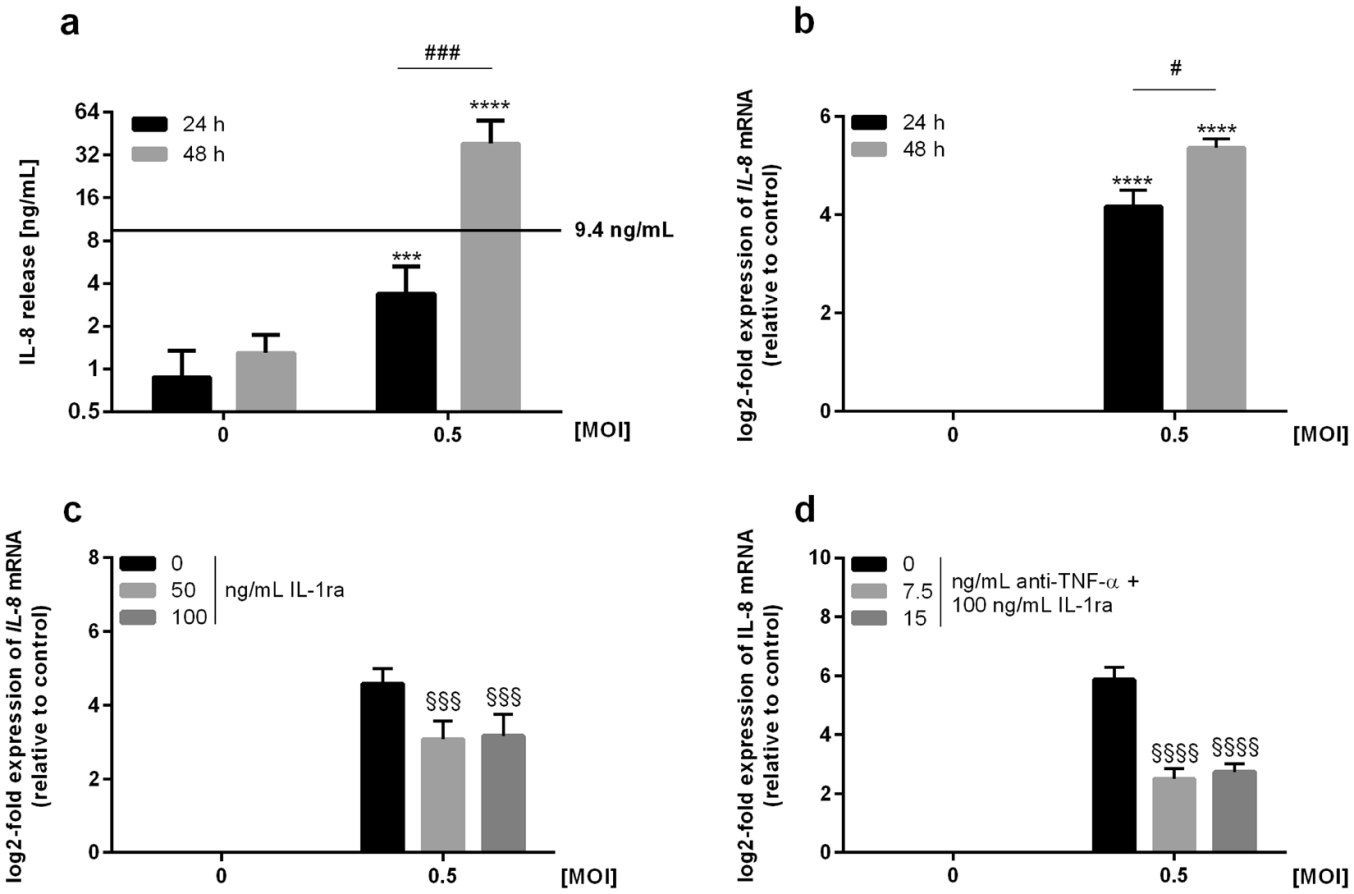
Infection of THP-1 cells with *L*. *pneumophila* induces paracrine activation of co-cultured lung epithelial cells. (**a**,**b**) THP-1 cells were stimulated with *L*. *pneumophila* (MOI 0.5) for 24 or 48 h. (A) IL-8 secretion of co-cultures was measured by ELISA; black line: cytokine release of THP-1 cells 48 h post infection calculated according to Fig. 1. (**b**) *IL-8* expression of co-cultured A549 cells was analysed by RT-qPCR. (**a**,**b**) Data are shown as mean ± SEM (n = 4). (**c**,**d**) Co-cultures were pre-incubated either with (**c**) IL-1ra (50 or 100 ng/mL) or (**d**) anti-TNF-α (7.5 or 15 ng/mL) together with 100 ng/mL IL-1ra for 1 h. Subsequently, THP-1 cells were stimulated with *L*. *pneumophila* (MOI 0.5) for 48 h. *IL-8* expression of A549 cells was analysed by RT-qPCR. Data are shown as mean ± SEM (n = 3). 2-Way ANOVA was performed with Sidak’s multiple comparison test as described: *Compared to corresponding control, ^#^compared to equally treated 24 h sample, ^§^compared to matching *L*. *pneumophila*-infected sample; ^#^p ≤ 0.05, ***or ^###^or ^§§§^p ≤ 0.001, ****or ^§§§§^p ≤ 0.0001, ns = not significant.

Assuming a cytokine-based intercellular communication between macrophages and alveolar epithelial cells, we determined the cytokine release of A549 cells co-cultured with *L*. *pneumophila*-infected THP-1 cells in a transwell system. Secretion analysis demonstrated a significant IL-8 release from co-cultures 48 hours post infection (Fig. 2a). Similarly, IL-6 secretion was induced under co-culturing conditions, while the release of IL-1β remained unaltered and TNF-α levels even decreased (Supplementary Fig. S3a–c). Next, we investigated whether the increased cytokine release of epithelial cells in response to *L*. *pneumophila* infection of co-cultured macrophages could be traced to changes in mRNA expression. Expression analyses of A549 cells indicated a significant time-dependent induction of *IL-8* following *L*. *pneumophila* infection of co-cultured THP-1 cells (Fig. 2b). Erroneous induction of *IL-8* expression through *L*. *pneumophila* contamination of alveolar epithelial cells was excluded (Supplementary Fig. S4).

To examine whether induction of *IL-8* was mediated by macrophage-derived IL-1β and TNF-α, co-cultures were treated with specific inhibitors blocking IL-1β and TNF-α signalling prior to *L*. *pneumophila* infection of macrophages and subsequent expression analyses of epithelial cells. *L*. *pneumophila*-induced expression of *IL-8* in A549 cells significantly decreased upon previous blocking the receptor for IL-1 (Fig. 2c) and was further diminished by the addition of an anti-TNF-α blocking antibody (Fig. 2d) confirming the IL-1β- and TNF-α-dependent pro-inflammatory activation of epithelial cells. The anti-TNF-α blocking antibody was only used in combination with the receptor antagonist of IL-1β, as the amount of TNF-α released by THP-1 cells was alone unable to induce *IL-8* expression in A549 cells (Figs 1c and S2b). The reduction of epithelial *IL-8* expression and thus its re-inducibility was investigated by continuing the incubation of lung epithelial cells after removal of initially co-cultured *L*. *pneumophila*-infected macrophages. Expression analysis of A549 cells demonstrated a reduction of *IL-8* expression to basal levels in response to the removal of infected macrophages (Supplementary Fig. S5).

### Paracrine mechanisms render alveolar epithelial cells hypo-responsive to *L*. *pneumophila* infection, but not to TNF-α exposure

**Figure 3:**
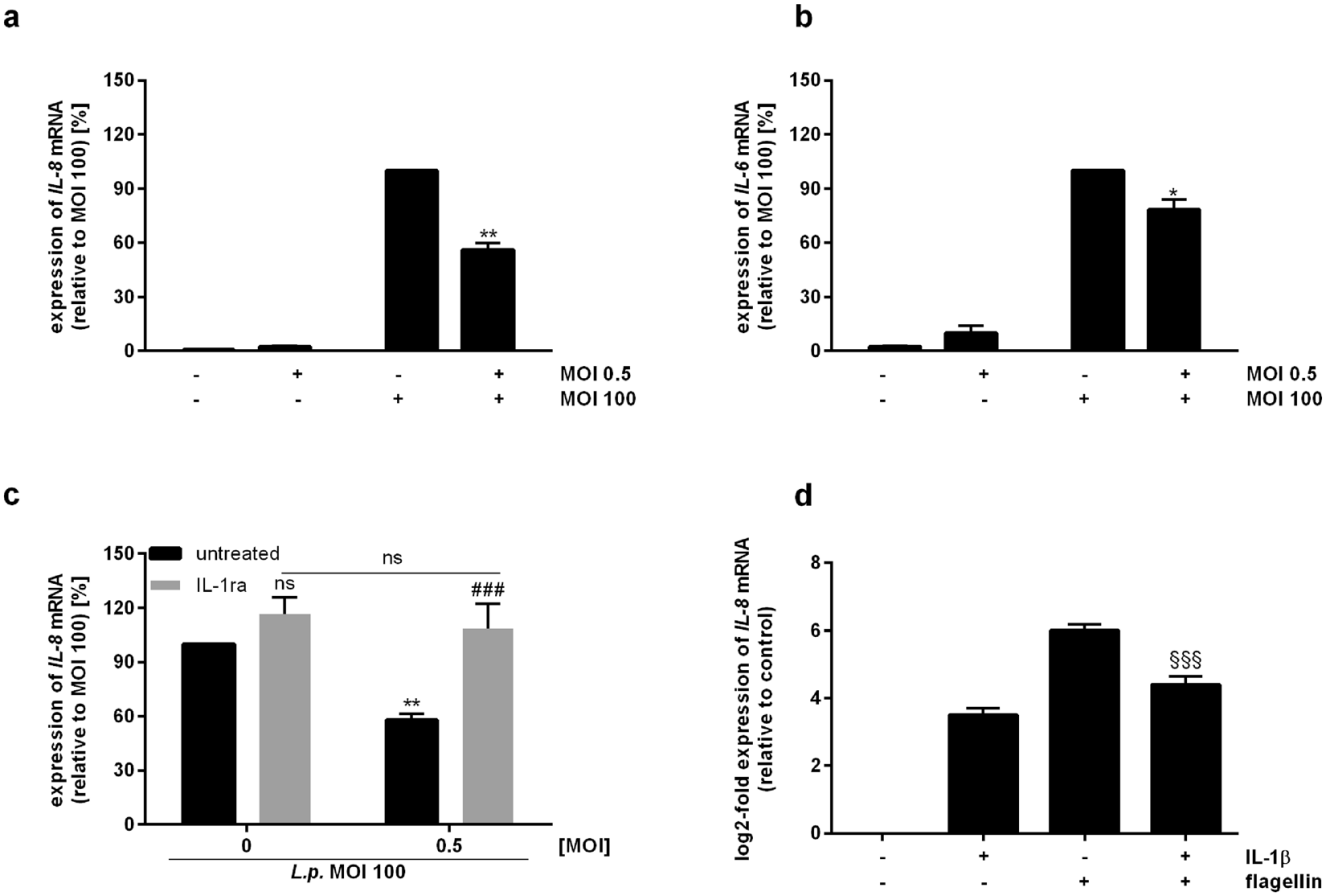
Paracrine mechanisms render alveolar epithelial cells hypo-responsive to *L*. *pneumophila* infection. (**a**–**c**) THP-1 cells were stimulated with *L*. *pneumophila* (MOI 0.5) for 48 h. Upon their removal, A549 cells were incubated in fresh medium (4 h) followed by *L*. *pneumophila* infection (MOI 100) for 3 h. Expression of (**a**,**c**) *IL-8* or (**b**) *IL-6* was analysed by RT-qPCR. (**c**) Co-cultures were stimulated with IL-1ra (100 ng/mL) for 1 h prior to re-stimulation. (**a**–**c**) Data are shown as average percentage ± SEM of individual pro-inflammatory mRNA induction relative to *L*. *pneumophila* (MOI 100) treatment (n = 4). (**d**) A549 cells were stimulated with IL-1β (1 ng/mL) for 2 h. Following medium renewal (4 h incubation), A549 cells were stimulated with flagellin (100 ng/mL) for 3 h. *IL-8* expression was analysed by RT-qPCR. Data are shown as mean ± SEM (n = 4). Two-tailed Student’s t-test (**a**,**b**,**d**) or 2-Way ANOVA with Sidak’s multiple comparison test (**c**) were performed as described: (**a**–**c**) *compared to *L*. *pneumophila*-infected sample (MOI 100), ^#^compared to corresponding IL-1ra-untreated sample; *p ≤ 0.05, **p ≤ 0.01, ^###^p ≤ 0.001, ns = not significant. (**d**) ^§^Compared to flagellin stimulation; ^§§§^p ≤ 0.001.

Hypothesizing that *L*. *pneumophila*-infected macrophages affect the responsiveness of lung epithelial cells to subsequent pathogen stimulation, we investigated the expression of pro-inflammatory cytokines upon re-stimulation of previously co-cultured epithelial cells. *L*. *pneumophila* infection of A549 cells antecedently co-cultured with uninfected THP-1 cells strongly induced *IL-8* expression (Fig. 3a). In contrast, infection of A549 cells previously co-cultured with *L*. *pneumophila*-infected macrophages led to a significant decrease in *IL-8* mRNA levels (Fig. 3a). The cytotoxic effect of prolonged exposure of THP-1 cells to *L. pneumophila* is negligible, as demonstrated by LDH assay (Fig. S14). The hypo-responsiveness of A549 cells towards re-stimulation was also observed for *IL-6* expression (Fig. 3b).

To investigate whether blocking of IL-1β signalling affects the response of A549 cells to a subsequent bacterial stimulus, we analysed *IL-8* expression in re-stimulated A549 cells co-cultured with untreated or *L*. *pneumophila*-infected THP-1 cells upon blocking the IL-1 receptor in both cell types (Fig. 3c). Following *L*. *pneumophila* infection of A549 cells, *IL-8* expression increased compared to co-cultures not blocked in IL-1β signalling. However, the induction of *IL-8* expression was only significant using *L*. *pneumophila*-infected THP-1 cells prior to re-stimulation (Fig. 3c), thereby implying a deterministic role of THP-1-derived IL-1β for rendering A549 cells hypo-responsive towards *L*. *pneumophila* infection.

For further examination of the hypo-responsiveness of epithelial cells, we established a co-culture-independent re-stimulation system using IL-1β and the flagellar protein flagellin as first and second stimulus, respectively. Expression analyses demonstrated that individual stimulation of A549 cells either with IL-1β or in particular with flagellin induced *IL-8* expression, respectively (Fig. 3d). In accordance with the results based on our co-culture system, re-stimulation of IL-1β-treated A549 cells with flagellin resulted in a significantly reduced *IL-8* induction in comparison to the flagellin stimulation of not pre-treated A549 cells (Fig. 3d,a). In contrast, following TNF-α treatment, re-stimulation with flagellin did not significantly reduce *IL-8* mRNA levels, thereby demonstrating that TNF-α is not involved in rendering A549 cells hypo-responsive to subsequent *L*. *pneumophila* infection (Supplementary Fig. S6a). Moreover, using TNF-α as second stimulus subsequent to IL-1β treatment expression of *IL-8* did not decrease but was significantly induced (Supplementary Fig. S6b).

In order to substantiate our findings, we analysed the induction of hypo-responsiveness in primary human aleveolar epithelial type 2 (AECII) cells. We found that, in analogy to the findings in A549 cells, primary AECII cells react with reduced *IL-8* expression when exposed to flagellin following pre-treatment with IL-1β vs. no pre-treatment (Fig. S7).

Taken together, our results demonstrated that, depending on IL-1β signalling, macrophages can render alveolar epithelial cells hypo-responsive to subsequent pathogen stimulation, thereby providing a mechanism to avoid overwhelming inflammation.

### Hypo-responsiveness is mediated by IκBα-regulated p65 translocation and is accompanied by changes in IL-8 mRNA half-life

**Figure 4:**
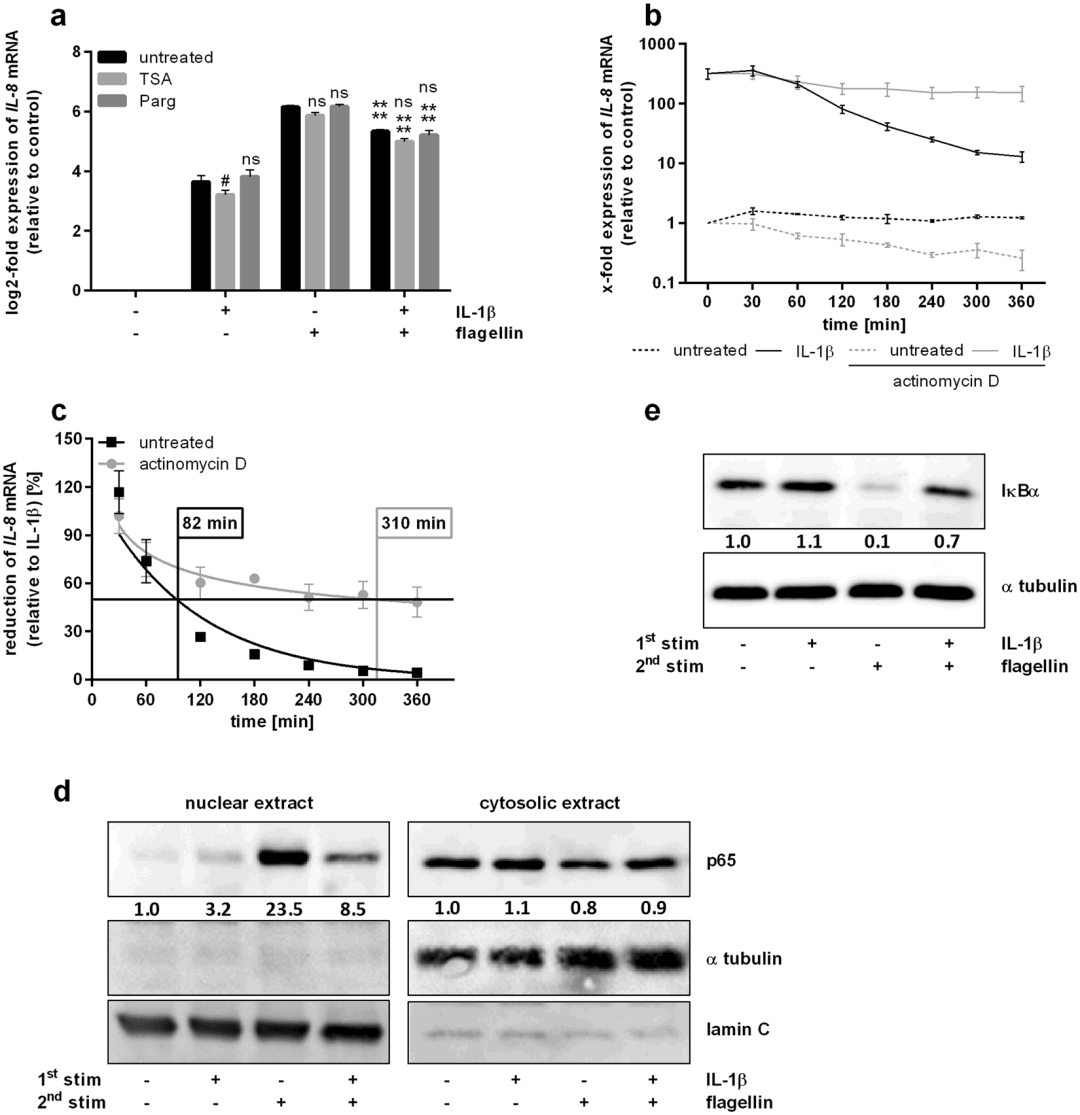
Hypo-responsiveness of lung epithelial cells is accompanied by changes in RNA half-life and IκBα-regulated nuclear presence of p65. (**a**) A549 cells were incubated with TSA (50 nM) or Parg (3 µM) for 2 h prior to stimulation with IL-1β (1 ng/mL, 2 h), medium renewal (4 h) and flagellin stimulation (100 ng/mL, 3 h). *IL-8* expression was analysed by RT-qPCR. Data are shown as mean ± SEM (n = 3). (**b**–**e**) A549 cells were stimulated with IL-1β (1 ng/mL, 2 h) followed by medium renewal. (**b**,**c**) Then, A549 cells were incubated with actinomycin D (10 µM, up to 360 min). (**b**) *IL-8* expression was analysed by RT-qPCR. (**c**) Data from B are shown as mean percentage ± SEM of individual *IL-8* induction relative to IL-1β stimulation (n = 4). *IL-8* half-life was calculated using non-linear regression. (**d**,**e**) After 4 h of incubation, A549 cells were stimulated with flagellin (100 ng/mL, 30 min). Nuclear and cytosolic presence of p65, α tubulin, and lamin C (**d**) and protein levels of IκBα and α tubulin (**e**) were analysed by western blotting and quantified. One out of three representative blots is shown. 2-Way ANOVA was performed with Sidak’s multiple comparison test as described: (**a**) *Compared to corresponding flagellin-stimulated sample, ^#^compared to corresponding untreated sample; ^#^p ≤ 0.05, ****p ≤ 0.001, ns = not significant.

Next, we investigated the mechanisms underlying the hypo-responsiveness of lung epithelial cells in more detail. Since pro-inflammatory gene expression is known to be associated with chromatin modifications, e.g. histone acetylation and methylation^24,25^, we examined whether those modifications affect *IL-8* mRNA levels upon re-stimulation of epithelial cells. Therefore, either class I and II histone deacetylases (TSA) or the lysine-specific histone demethylase 1 (LSD1; Parg) were inhibited prior to re-stimulation of A549 cells. In accordance with our co-culturing conditions, expression analysis upon re-stimulation of untreated alveolar epithelial cells demonstrated a significant decrease of *IL-8* levels compared to single flagellin treatment (Figs 3a and 4a). However, following re-stimulation, neither blocking of histone deacetylation nor of LSD-1 affected *IL-8* expression (Fig. 4a).

We hypothesized that mRNA stability is affected by the primary stimulus IL-1β. Initial expression analysis of A549 cells demonstrated that *IL-8* mRNA levels were strongly induced by IL-1β stimulation and decreased within 4 h after removing the stimulus (Fig. 4b). In a next step, we inhibited global transcription by actinomycin D after *IL-8* mRNA induction and observed an only moderate *IL-8* mRNA decrease over time (Fig. 4b). Calculating the half-life of *IL-8* mRNA following IL-1β stimulation by non-linear regression, we found an almost four-fold extended half-life in A549 cells after blocking global transcription (Fig. 4c). This indicates that *de novo* expression of certain genes is able to reduce *IL-8* mRNA stability and might be involved in the generation of a hypo-responsive state subsequent to bacterial infection.

Furthermore, we analysed the nuclear presence of NF-κB p65 subunit (p65) upon re-stimulation of epithelial cells. Western Blot analysis of nuclear fractions indicated a strong signal for p65 in response to flagellin stimulation of A549 cells (Fig. 4d). In contrast, re-stimulation with flagellin subsequent to IL-1β treatment resulted in a much weaker signal corresponding to the reduced expression of pro-inflammatory cytokines following re-stimulation of lung epithelial cells (Fig. 4d). To further verify the involvement of NF-κB signalling in triggering the hypo-responsiveness of epithelial cells, we determined protein levels of the negative regulator IκBα. Compared to IL-1β stimulation, Western Blot analysis of A549 cells revealed a significant flagellin-induced reduction of global IκBα (Fig. 4e). In contrast, flagellin re-stimulation following IL-1β treatment resulted in IκBα protein levels comparable to those upon single IL-1β stimulation, reflecting an inverse correlation to the expression of pro-inflammatory cytokines (Fig. 3a and b) and nuclear presence of p65 (Fig. 4d).

In summary, degradation of IκBα protein and consequently translocation of NF-κB/p65 were impaired by re-stimulation and subsequent to IL-1β treatment, thus confirming an involvement of NF-κB in triggering the hypo-responsiveness of pulmonary epithelial cells. Moreover, the reduced *IL-8* mRNA stability upon IL-1β stimulation might also contribute to the observed phenomenon.

### Kinetic modelling suggests a role for IRAK-1 in epithelial hypo-responsiveness

**Figure 5:**
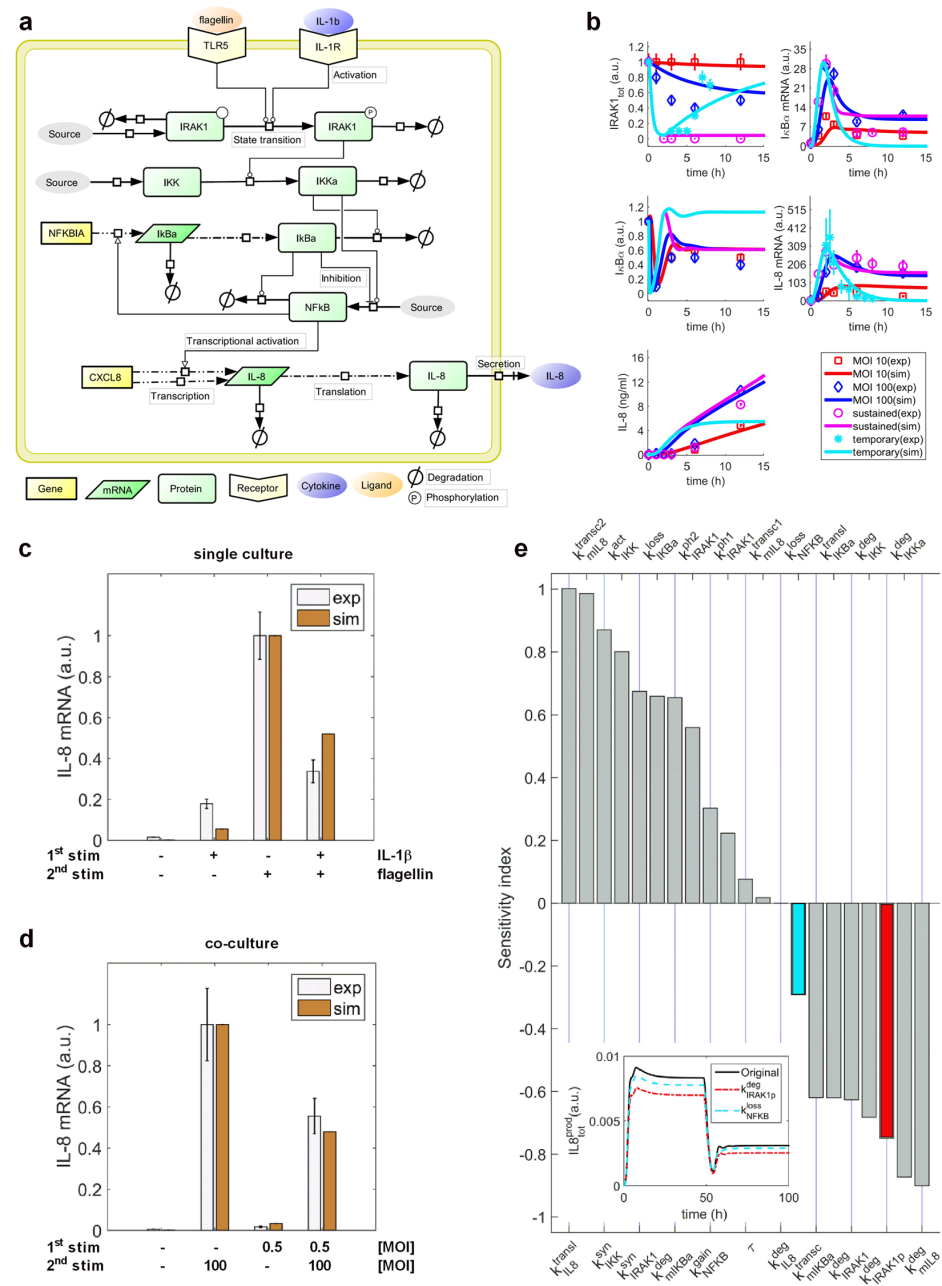
Mathematical modelling of the NF-κB signalling pathway. (**a**) Model scheme. (**b**) Model calibration. The model was fitted using time-series data: sustained challenge of lung epithelial cells with *L*. *pneumophila* (MOI 10 or MOI 100, up to 24 h); A549 cell stimulation with IL-1β (1 ng/mL) for up to 12 h (sustained) or for 2 h followed by medium renewal and further incubation for up to 24 h (temporary). Lines represent model simulations and the symbols account for experimental data (mean ± SD). (**c**,**d**) Model validation. The data-driven model possesses the ability to predict experimental data (mean ± SEM) showing the reduced *IL-8* mRNA expression of lung epithelial cells after pre-stimulation. (**c**) A549 cells were stimulated with IL-1β (1 ng/mL, 2 h) followed by medium renewal (4 h incubation) and flagellin stimulation (100 ng/mL, 3 h). (**d**) THP-1 cells were stimulated with *L*. *pneumophila* (MOI 0.5) for 48 h. Upon their removal, A549 cells were incubated in fresh medium (4 h) followed by *L*. *pneumophila* infection (MOI 100) for 3 h. (**e**) Sensitivity analysis. ${k}_{IRAK1}^{deg}$ and ${k}_{IRAK1p}^{deg}$ rank in top five parameters to negatively influence IL-8 production. For illustration, the inset figure shows the influence of ${k}_{IRAK1p}^{deg}$ (red) and ${k}_{NFkB}^{loss}$ (cyan) on IL-8 production after increasing their original values by 25%. ${k}_{IRAK1p}^{deg}$ has stronger impact on reduction of IL-8 production. a.u.: arbitrary unit. IL-1R: IL-1 receptor.

To further investigate the mechanism of IL-1β-induced NF-κB signalling on rendering alveolar epithelial cells hypo-responsive towards subsequent bacterial infection, we developed a kinetic model of ordinary differential equations. Briefly, the kinetic model accounts for the temporal dynamics of the core NF-κB signalling network during stimulation of pulmonary epithelial cells with *L*. *pneumophila*, flagellin or IL-1β. The latter two were used as surrogates either for *L*. *pneumophila* infection or the observed paracrine communication between alveolar macrophages and epithelial cells. This core module includes the signal mediators IRAK-1, IκB kinase (IKK), IκBα, the transcription factor NF-κB, and the pro-inflammatory cytokine IL-8, which was used as a surrogate for epithelial cell-mediated inflammatory response (Fig. 5a). To make our model predictive, we characterized model parameter values by fitting model simulations to time-series experimental data, which account for the dynamics of the network components after stimulating alveolar epithelial cells with *L*. *pneumophila* or IL-1β (Fig. 5b; see Materials and Methods for details). After calibrating the model with experimental data, we evaluated identifiability of model parameters (for details see Materials and Methods). The results showed that eight out of 14 estimated parameters were identifiable in the defined parameter interval (Supplementary Fig. S8). The data-driven model predicted a reduction of flagellin-induced *IL-8* expression as a result of lung epithelial cell pre-stimulation with IL-1β (Fig. 5c), that very closely resembles the experimental data (Fig. 3d). Similarly, the model correctly predicted the significant decrease in *L*. *pneumophila-*induced *IL-8* mRNA levels by previous co-culture of pulmonary epithelial cells with *L*. *pneumophila*-infected macrophages (Figs 3a and 5d).

Next, we used the data-driven model to identify molecules with strong impact on the hypo-responsiveness mechanism during *L*. *pneumophila* infection. To this end, we performed sensitivity analysis, an unbiased computational method that helps to identify important interactions of the NF-κB signalling network controlling the dynamics of IL-8 expression. As shown in Fig. 5e, the sensitivity indexes of IRAK-1 degradation (${k}_{IRAK1}^{deg}$) and phosphorylated IRAK-1 degradation (${k}_{IRAK \mbox{-} 1p}^{deg}$, highlighted in red) rank in the top five of model parameters that negatively affect IL-8 secretion. Thus, a perturbation in ${k}_{IRAK \mbox{-} 1p}^{deg}$ would extensively influence the temporal dynamics of IL-8 production compared to a parameter with a smaller sensitivity index value (${k}_{NF \mbox{-} kB}^{loss}$, highlighted in cyan). In addition, IRAK-1 is a kinase specifically involved in IL-1β and toll-like receptor (TLR) but not TNF-α signalling^26,27^. Taken together, the model predicted a strong impact of IRAK-1 on IL-8 production, suggesting its importance in regulating the hypo-responsiveness mechanism during *L*. *pneumophila* infection.

**Figure 6:**
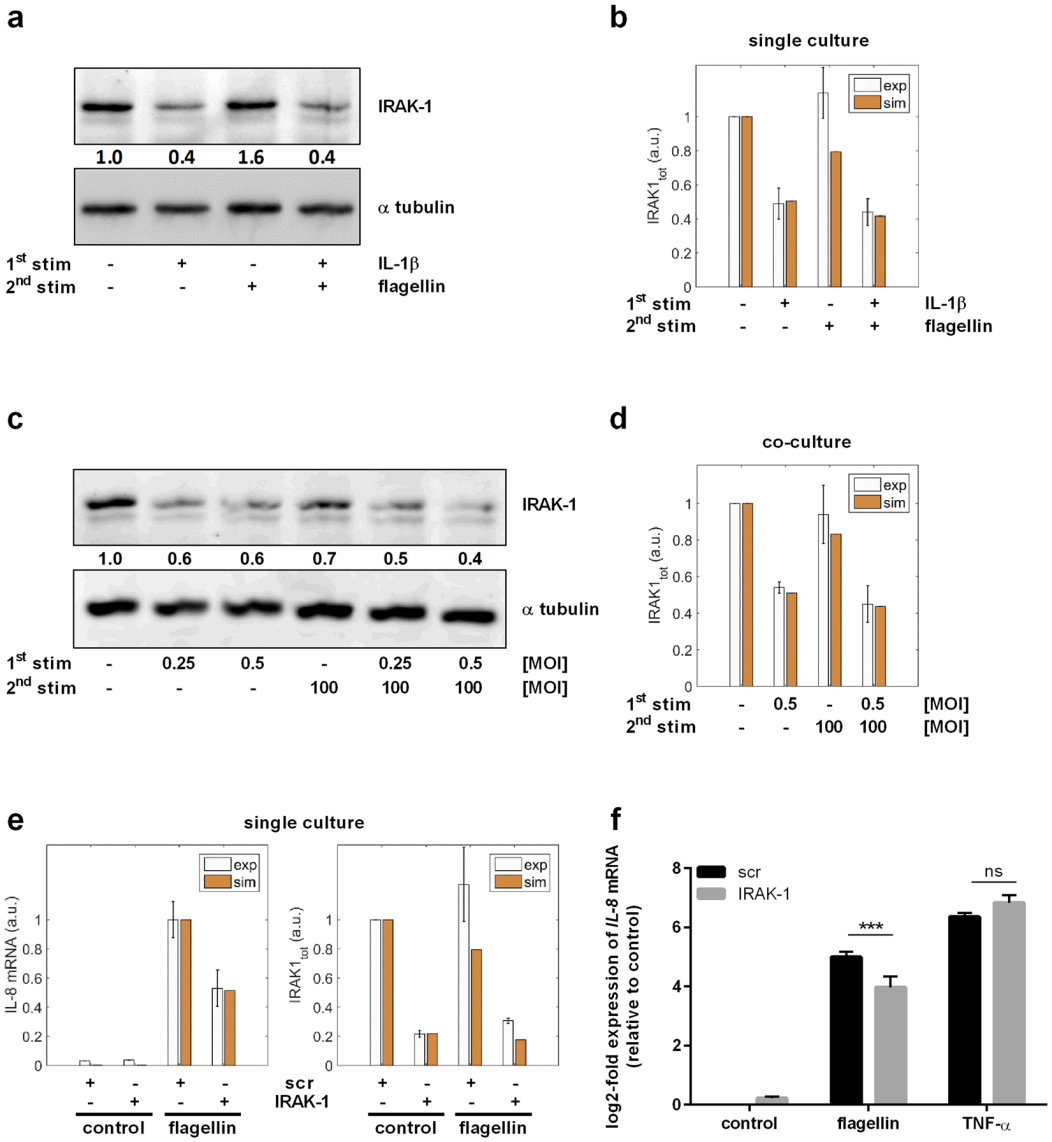
Hypo-responsiveness is mediated by IRAK-1 and can be mimicked by its knockdown. (**a**,**b**) A549 cells were stimulated with IL-1β (1 ng/mL, 2 h) followed by medium renewal (4 h incubation) and flagellin stimulation (100 ng/mL, 3 h). (**a**) Protein expression was analysed by western blotting and quantified. One out of four representative blots is shown. (**b**) The experimental data were used to validate model predictions. (**c**,**d**) THP-1 cells were stimulated with *L*. *pneumophila* (MOI 0.25 or 0.5) for 48 h. Upon their removal and subsequent medium renewal (4 h incubation), A549 cells were stimulated with *L*. *pneumophila* (MOI 100, 3 h). (**c**) Protein levels of IRAK-1 and α tubulin were analysed by western blotting and quantified. One out of four representative blots is shown. (**d**) Model predictions were validated using experimental data. (**e**,**f**) A549 cells were transfected with control (scr) or IRAK-1 siRNA (48 h) and stimulated with flagellin (100 ng/mL) or TNF-α (100 ng/mL) for 3 h. (**e**) The model was used to predict *IL-8* expression upon IRAK-1 depletion, which was in accordance with the experimental data. (**f**) *IL-8* expression was analysed by RT-qPCR and normalized to an untreated scr control (mean ± SEM, n = 4). (**e**) The data were compared with model predictions. Statistical analysis: (**f**) 2-Way ANOVA with Sidak’s multiple comparison test as described; *Compared to corresponding scr, ***p ≤ 0.001; ns = not significant.

To prove this model prediction, we analysed IRAK-1 protein levels under different conditions and in the re-stimulation setting both *in silico* and experimentally: Western Blot analysis of A549 cells showed a significant reduction of IRAK-1 expression upon IL-1β stimulation, whereas single treatment with flagellin did not affect IRAK-1 levels (Fig. 6a). In accordance with single IL-1β treatment, subsequent re-stimulation with flagellin resulted in significantly reduced IRAK-1 levels, reflecting the reduced expression of pro-inflammatory cytokines following re-stimulation of epithelial cells. This was in agreement with the model’s predictions (Fig. 6b). Confirming the results of the re-stimulation system, Western Blot analysis upon *L*. *pneumophila* infection of A549 cells previously co-cultured with *L*. *pneumophila*-infected THP-1 cells demonstrated an IL-1β-dependent reduction of IRAK-1 levels (Fig. 6c). Again, the model’s predictions were consistent with the data (Fig. 6d), proving its ability to predict the dynamics of NF-κB signalling in alveolar epithelial cells during *L*. *pneumophila* infection. Contrary to the IL-1β stimulation and the co-culture system, but in line with the previously determined *IL-8* mRNA expression, TNF-α treatment alone or prior to flagellin stimulation did not affect IRAK-1 protein levels (Supplementary Fig. S9).

As microRNA-146a (miR-146a) has been described to be a negative regulator of IRAK-1^28^, we analysed its involvement in triggering the hypo-responsiveness of alveolar epithelial cells towards subsequent bacterial infection. Expression analyses of A549 cells demonstrated a strong induction of miR-146a upon IL-1β stimulation whereas single treatment with flagellin only weakly affected miR-146a levels (Supplementary Fig. S10a). However, in accordance with single IL-1β treatment, subsequent re-stimulation with flagellin significantly induced miR-146a expression, suggesting a miR-146a-dependent reduction of IRAK-1 levels following re-stimulation of epithelial cells. Next, we investigated the effect of miR-146a overexpression on *IRAK-1* and *IL-8* mRNA levels in response to subsequent flagellin stimulation. A549 cells’ expression analyses indicated a concentration-dependent overexpression of miR-146a that, however, was not affected by flagellin treatment (Supplementary Fig. S10b). *IRAK-1* mRNA levels inversely correlated with miR-146a overexpression (Supplementary Fig. S10c). In line with the re-stimulation settings (Fig. 6a), flagellin treatment was unable to further decrease the expression of *IRAK-1* (Supplementary Fig. S10c). Moreover, upon adjusting miR-146a to physiological levels, *IRAK-1* expression was not affected anymore. In addition, flagellin-induced expression of *IL-8* was inversely correlated with miR-146a levels, whereas RNA levels of miR-146a similar to physiological concentrations no more reduced, but rather induced *IL-8* expression (Supplementary Fig. S10d). This is in accordance with the model simulations that predicted a hypo-responsiveness of lung epithelial cells to re-stimulation is not regulated by miR-146a (Figs 5c,d and 6b,d,e).

Taken together, our results corroborated that the IL-1β-induced degradation of IRAK-1 in lung epithelial cells can negatively impact *IL-8* expression upon infection by *L*. *pneumophila*, suggesting its crucial role in regulating hypo-responsiveness. Furthermore, miR-146a seems not to be involved in rendering pulmonary epithelial cells hypo-responsive towards subsequent bacterial infection.

### Hypo-responsiveness can be mimicked by IRAK-1 knockdown

We further investigated the effect of IRAK-1 on *IL-8* expression *in silico* and *in vitro*: model simulations predicted a strongly reduced flagellin-mediated *IL-8* induction of alveolar epithelial cells upon IRAK-1 depletion (Fig. 6e). Thus, A549 cells were transiently depleted for IRAK-1 protein by siRNA and subsequently stimulated with flagellin or TNF-α, respectively. Expression analysis indicated a significant reduction of *IL-8* mRNA levels upon depletion of IRAK-1 followed by flagellin stimulation (Fig. 6f). In contrast, stimulation with TNF-α rather enhanced *IL-8* expression in response to IRAK-1 depletion. Knockdown efficiencies were predicted (Supplementary Fig. S11a), as well as verified on RNA (Supplementary Fig. S11b) and protein levels (Supplementary Fig. S11c).

**Figure 7:**
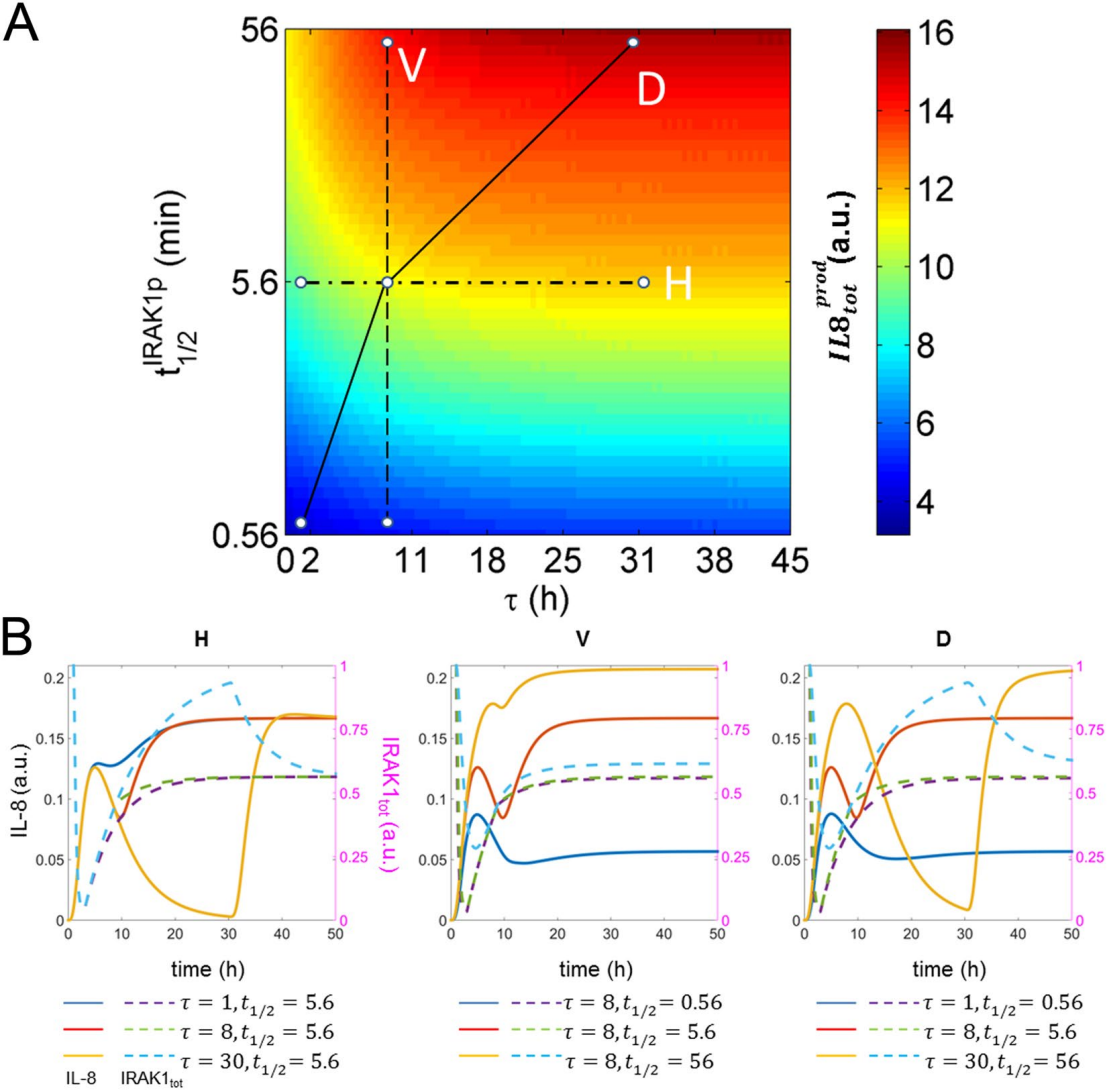
*In silico* modelling of lung epithelial cell hypo-responsiveness to re-stimulation. (**A**) Overall production of IL-8 ($IL{8}_{tot}^{prod}$) for different combined values of *τ* and ${t}_{1/2}^{IRAK1p}$ is shown. *τ* represents the interval between the two stimuli: IL-1β and flagellin. ${t}_{1/2}^{IRAK1p}$ denotes for the half-life of phosphorylated IRAK-1, and its wild type value is 5.6 min. (**B**) Temporal dynamics of IL-8 (*IL8*; solid lines) and total amounts of IRAK-1 (*IRAK1*_*tot*_; dashed lines) are presented for different combinations of *τ* and ${t}_{1/2}^{IRAK1p}$. The selected combinations of *τ* and ${t}_{1/2}^{IRAK1p}$ (**H**,**V** and **D**) are delineated by different types of lines in a.

Finally, we used the kinetic model to analyse the effect of IRAK-1 half-life on the tolerance of alveolar epithelial cells to stimulation. To this end, we designed an *in silico* experiment, which imitates the co-culture setting using different half-lives of phosphorylated IRAK-1 (${t}_{1/2}^{IRAK1p}$) and time intervals *τ* between the two stimuli. Specifically, perturbation of ${t}_{1/2}^{IRAK1p}$ accounts for potential interindividual variability in IRAK-1 stability, and modulation of *τ* represents different time intervals between the first stimulation of lung epithelial cells by macrophage-secreted IL-1β and their further activation by direct *L*. *pneumophila* contact. We perturbed ${t}_{1/2}^{IRAK1p}$ and *τ* and computed the total IL-8 secretion by alveolar epithelial cells ($IL{8}_{tot}^{prod}$; Fig. 7A). In addition, several combinations of *τ* and ${t}_{1/2}^{IRAK1p}$ values were selected to show the corresponding temporal dynamics of IL-8 and IRAK-1 (Fig. 7B). As shown in the figure, increasing *τ* positively enhances $IL{8}_{tot}^{prod}$ (Fig. 7A). This is due to the recovery of IRAK-1 levels after temporary IL-1β stimulation (Fig. 7B–H). Increase of ${t}_{1/2}^{IRAK1p}$ results in higher $IL{8}_{tot}^{prod}$ (Fig. 7A) due to IRAK-1 stabilization (Fig. 7B–V). Moreover, simultaneous increase of both ${t}_{1/2}^{IRAK1p}$ and *τ* results in a more significant increase of $IL{8}_{tot}^{prod}\,$(Fig. 7A) due to higher peak and steady state levels of IL-8 (Fig. 7B–D).

In summary, the model simulations correctly predicted the impact of IRAK-1 in the experimentally observed hypo-responsiveness of lung epithelial cells during of *L*. *pneumophila* infection and further elaborated on the mechanism.

## Discussion

Innate immunity plays an important role in host defense against bacterial pneumonia, but it has to be tightly controlled to prevent organ damage and development of sepsis. We found that *L*. *pneumophila*-infected macrophages provoke a pro-inflammatory activation of neighboring lung epithelial cells, but in addition render them hypo-responsive to direct infection with the same pathogen. By combining experimental studies and mathematical modelling, we identified a paracrine mechanism of macrophage-secreted IL-1β inducing a prolonged IRAK-1 degradation in lung epithelial cells. This suggests an important but ambivalent immunomodulatory role of macrophages in *L*. *pneumophila* infection.

Previous studies have shown that human macrophages are important target cells of *L*. *pneumophila* in lung infection, that they are permissive for legionella replication, and simultaneously secrete pro-inflammatory cytokines^29–31^. Accordingly, we found time- and concentration-dependent release of IL-1β, IL-6, IL-8, TNF-α and IL-10 from legionella-infected human macrophage-like cells. Similarly, alveolar epithelial cells that constitute the largest part of lung surface are activated by *L*. *pneumophila* ^32^.

To better understand the intra-alveolar crosstalk of macrophages and lung epithelial cells in legionella infection, we describe here a co-culture model of human macrophages and pulmonary epithelial cells for this pathogen. After infection of THP-1 cells, alveolar epithelial cells expressed and released pro-inflammatory cytokines. Considering a paracrine effect, we found that the activation of neighboring alveolar epithelial cells by infected macrophages depended on IL-1β. Accordingly, it has been shown in a comparable *in vitro* model as well as in a murine infection model for pneumococci infection, that macrophages prime lung epithelial cells by IL-1β to produce IL-8, or its murine paralogues, resulting in neutrophil recruitment in murine pneumonia^9^. Moreover, pneumococci-infected murine macrophages evoked pro-inflammatory gene expression in alveolar epithelial cells by type I interferons^33^.

Interestingly, in our model, these by-standing pulmonary epithelial cells showed a dampened IL-8 release upon subsequent direct infection with *L*. *pneumophila*. This attenuating effect on legionella-induced inflammation also depended on IL-1β. In contrast, the reactivity to stimulation with TNF-α was not impaired. Since IL-8 is known to induce neutrophil migration^34,35^, this phenomenon might be beneficial for host immunity, as it has been shown that neutrophil recruitment is on the one hand important for bacterial growth control but on the other hand can lead to the disruption of the alveolar barrier^36,37^, higher rates of bacteremia and early death in murine pneumonia^38^.

While a hypo-responsiveness of alveolar epithelial cells mediated by paracrine mechanisms has not been described by now, the induction of a similar phenomenon on single cells, the endotoxin tolerance of macrophages, has been intensively studied^39,40^. Among the variety of analysed mechanisms, transient chromatin-dependent silencing of pro-inflammatory genes, i.e. acetylation of histone H4 or trimethylation of histone H3 at lysine 4, has been described^41^. These silencing effects have been shown to be preventable or revertible by inhibition of histone-deacetylases by TSA or H3K4 demethylase LSD1 by Parg. However, both strategies did not influence the reduced reactivity of IL-1β-pre-stimulated lung epithelial cells, arguing against this chromatin-associated effect in our model.

Proceeding with the search for the underlying mechanisms of this specific epithelial hypo-responsiveness to *L*. *pneumophila*, we observed a reduced mRNA stability for *IL-8* after IL-1β stimulation that appear to depend on *de novo* gene expression. Wickremasinghe *et al*. obtained comparable results by TNF-α treatment of epithelial cells^42^. It has previously been shown that IL-8 expression can be regulated at the level of mRNA stability, e.g. depending on KH-type splicing regulatory protein^43^ and tristetraprolin^44^. This mechanism could explain our observation of a transcription-dependently reduced mRNA stability for *IL-8* upon IL-1β stimulation. In addition, we hypothesized that pre-transcriptional mechanisms might be involved and observed that pre-exposure with IL-1β strongly reduced flagellin-induced IκBα degradation and nuclear translocation of p65 upon re-stimulation. Several mechanisms have been described to negatively influence canonical NF-κB activation^45,46^.

To analyse the mechanism of hypo-responsiveness in more detail, we constructed a kinetic model accounting for the activation of the NF-κB signalling pathway in lung epithelial cells by infection with *L*. *pneumophila*, direct stimulation with flagellin or IL-1β. The model could predict the fine-tuned regulation of NF-κB signalling during *L*. *pneumophila* infection of alveolar epithelial cells. With the help of sensitivity analysis, we identified the potential importance of IRAK-1 stability on regulating the hypo-responsiveness of pulmonary epithelial cells to *L*. *pneumophila* infection.

IRAK-1 is a downstream mediator of IL-1R/TLR but not of TNF-α^26,27^. We observed a long-lasting degradation after pre-stimulation with IL-1β but not TNF-α. In accordance with the prediction by the data-driven model, knockdown of IRAK-1 and subsequent stimulation with flagellin showed a reduced *IL-8* mRNA expression also comparable to results obtained for re-stimulation. This goes in line with reports by Li *et al*.^26^ and Siedlar *et al*.^47^ showing involvement of IRAK-1 in either bacterial lipoprotein or Pam3Cys mediated tolerance in monocytic cell lines. Furthermore, it was reported that a reversion of bacterial lipoprotein induced tolerance was achieved by IRAK-1 overexpression^48^. It was demonstrated that IRAK-1 expression can be directly influenced by miR-146a^28^. Indeed, miR-146a was induced in lung epithelial cells, both after paracrine IL-1β exposure or direct legionella infection. Artificial overexpression of this miRNA reduced *IRAK-1* mRNA levels and thus TLR-signalling-mediated IL-8 expression, however only in very high concentrations, arguing against miR-146a as the underlying effector. Accordingly, TLR-induced tolerance in murine alveolar epithelial cells was shown not to depend on miR-146a^49^. However, several other immunologically relevant targets of miR-146a have been validated so far (see miRTarBase^50^), so that a miR-146a effect cannot be ruled out.

**Figure 8:**
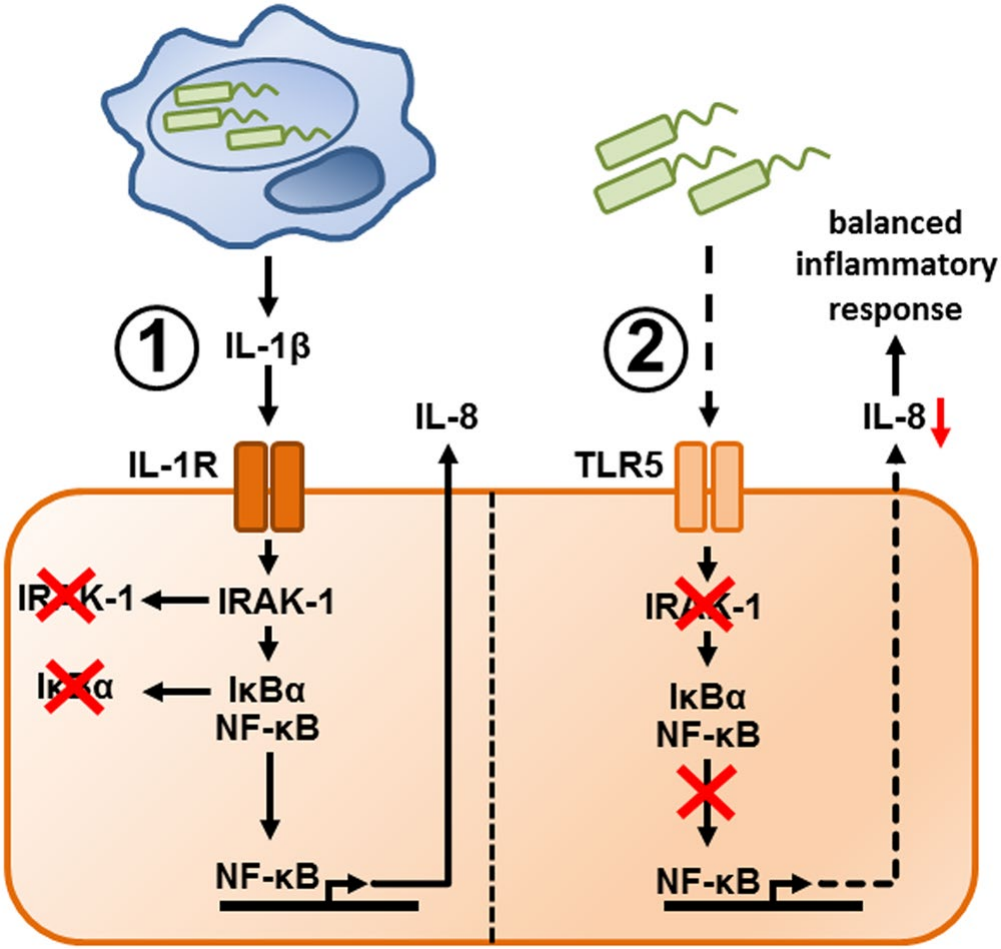
Macrophages can induce hypo-responsiveness of epithelial cells to *L*. *pneumophila* infection through a paracrine mechanism. Macrophages (blue) induce tolerance of neighboring lung epithelial cells (orange) to *L*. *pneumophila* (green) infection through an IL-1β-dependent mechanism (①), which results in prolonged depletion of IRAK-1 in the epithelial cells (②). This mechanism attenuates subsequent pro-inflammatory activation of these epithelial cells upon TLR5-dependent recognition of *L*. *pneumophila* and thus prevents potentially harmful inflammatory reactions in the lung.

Taken together, our experiments and model simulations indicate that alveolar macrophages can negatively regulate the responsiveness of neighboring lung epithelial cells to bacterial infection by the release of IL-1β, resulting in a long lasting downregulation of IRAK-1 and a reduced *IL-8* mRNA stability (Fig. 8). By preventing excessive local secretion of pro-inflammatory cytokines (i.e. cytokine storm) and thereby negatively regulating the recruitment of immune cells (mainly neutrophils) to the site of infection, the intercellular communication may be useful for avoiding an overwhelming inflammatory response. Interestingly, a variant of IRAK-1 with greater auto-phosphorylation (i.e. more stable phosphorylated form) has been associated with increased NF-κB activation and inflammatory responses in bacteria challenged cells^51,52^. Moreover, clinical studies indicated that polymorphisms affecting the stability of IRAK-1 can modulate the intensity of the inflammatory response during acute inflammatory episodes^53^. These experimental findings are consistent with our model simulation supporting the conclusion that interindividual variability in IRAK-1 stability can change the intensity of the pro-inflammatory response triggered by alveolar epithelial cells upon *L*. *pneumophila* infection.

We anticipate that the systems biology strategy applied in this study will increase our insight into intercellular communication during infection and be applicable in a wide range of biomedical systems.

## Materials and Methods

### Chemicals and antibodies

Ham’s F12 medium was obtained from GE Healthcare Europe (Freiburg, Germany). RPMI-1640, GlutaMax and FCS were purchased from Life Technologies (Darmstadt, Germany). PBS was acquired from Biochrom (Berlin, Germany). Pargyline (Parg), phorbol 12-myristate 13-acetate (PMA) and trichostatin A (TSA) were supplied by Sigma-Aldrich Chemie (Munich, Germany). IL-1β, IL-1ra, TNF-α and anti-TNF-α were obtained from R&D Systems (Wiesbaden, Germany). Flagellin from *Salmonella typhimurium* was purchased from InvivoGen (San Diego, USA) and actinomycin D was supplied from Biovision (Milpitas, USA). All other applied chemicals were of analytical grade and acquired from commercial sources.

### *L*. *pneumophila* culture

*L*. *pneumophila* Corby wild type (provided by Prof. Dr. K. Heuner, RKI, Berlin, Germany) was routinely grown on buffered charcoal-yeast extract (BCYE) agar plates at 37 °C for three days. Then, bacterial material was inoculated in an appropriate volume of PBS and optical density at 600 nm (OD600) was determined by an Ultrospec 10 Cell Density Meter (GE Healthcare Europe, Freiburg, Germany). An OD600 of one corresponded to 2 * 10^9^ bacteria/mL. The desired multiplicity of infection (MOI) was obtained by serial dilutions in PBS and lastly, in cell specific medium.

### Cell culture and co-culture

The alveolar type II cell line A549 and the monocytic cell line THP-1 were purchased from Sigma-Aldrich (Munich, Germany). A549 cells were cultured using Ham’s F12, and THP-1 cells were cultivated in RPMI-1640, both supplemented with GlutaMax and 10% FCS, in a humidified incubator at 37 °C and 5% CO_2_. A549 cells were either seeded reaching a cell density of 1.2 * 10^5^ cells/cm^2^ at the day (single stimulation) or at the end of the experiment (co-culture). Using 20 nM PMA for 24 h, THP-1 cells were differentiated towards macrophage-like phenotype and subsequently seeded at 8 * 10^4^ cells/cm^2^. Stimulation was performed using varying concentrations of IL-1β or TNF-α for the indicated times.

Co-culture of PMA-treated THP-1 and A549 cells was established using translucent ThinCert Cell Culture Inserts with a pore size of 0.4 µm (Frickenhausen, Germany) in Ham’s F12 with GlutaMax and 10% FCS. The insert contained PMA-treated THP-1 cells. Separated cell containing compartments were cultured in a humidified incubator at 37 °C and 5% CO_2_ and assembled at the date of infection.

### Transfection

A549 cells were subjected to siRNA-mediated *IRAK-1* mRNA knockdown (Silencer Select siRNA; Life Technologies) as well as miR-146a-5p overexpression (mirVana miRNA Mimic, Life Technologies) or a corresponding scrambled (scr), non-targeting nucleic acid negative control (Silencer Select Negative Control No. 1 siRNA and mirVana  miRNA Mimic Negative Control No. 1, respectively). The reverse transfections were performed according to the manufacturer’s recommendation. After total incubation of 24 h (mirVana miRNA Mimics) or 48 h (Silencer Select siRNAs), medium renewal was performed and A549 cells were stimulated as indicated.

### Stimulation and *L*. *pneumophila* infection

Stimulation of A549 cells was performed using varying concentrations of IL-1β or TNF-α for the indicated times. *L*. *pneumophila* (MOI 0.5) was prepared as described above and added to the THP-1 cells for 24 and 48 h, respectively.

In the co-culture system, PMA-treated THP-1 cells were infected with *L*. *pneumophila* (MOI 0.5) in the upper compartment for 24 and 48 h. If indicated, co-cultured cells were pre-treated with either IL-1ra or IL-1ra together with anti-TNF-α for 1 h.

For re-stimulation, A549 cells were first exposed to IL-1β or TNF-α for 2 h, washed three times with PBS, and incubated for further 4 h with fresh medium. In the second step, A549 cells were stimulated with flagellin or TNF-α for the specified time. Where indicated, A549 cells were pre-treated with TSA or Parg for 2 h. Where indicated, AECII cells were used following the same protocol.

For re-stimulation in the co-culture system, PMA-treated THP-1 cells in the upper compartment were infected with *L*. *pneumophila* (MOI 0.5) for 48 h. Then, the transwell inserts were removed and A549 cells in the lower compartment were washed three times with PBS and incubated for 4 h with fresh medium. Finally, A549 cells were infected with a MOI of 100 for additional 3 h. Where indicated, both compartments were pre-treated with IL-1ra for 1 h.

### CFU assay

Input controls of used MOIs were routinely performed by serial diluting and plating on BCYE agar plates, which were also used for determination of colonies in the upper compartment. For colony forming unit (CFU) counting of the lower compartment, supernatant was collected and plated without diluting. After three days of growing at 37 °C, CFUs were quantified and MOI was calculated.

### ELISA and Multiplex Luminex Assay

Cell-free supernatants of THP-1 cells or co-cultures were measured using either the BD OptEIA for IL-8 (BD Biosciences, Heidelberg, Germany) or the Luminex Performance Human High Sensitivity Cytokine Magnetic Panel A (IL-1β, IL-6, IL-10 and TNF-α; R&D Systems GmbH, Wiesbaden-Nordenstadt, Germany). Both were performed according to the manufacturer’s specifications. Range of detection for each molecule were [pg/ml]: 3.1–200 (IL-8), 0.292–1 (IL-1β), 0.952–3 (IL-6), 0.464–1 (IL-10), 0.757–3 (TNF-α). Samples were diluted accordingly. For measurement, Infinite® M200 Pro microplate reader (Tecan, Maennedorf, Switzerland) and MAGPIX System (Luminex, Austin, USA) were used.

### Western Blot

For determination of IκBα, IRAK-1 and p65 protein expression, A549 cells were stimulated or infected as indicated, washed twice with PBS and total lysates (IκBα, IRAK-1) or nuclear and cytosolic extracts (p65) were separated by SDS-PAGE. Blotting and immobilization was performed with a Protran 0.2 µm Nitrocellulose (GE Healthcare Europe) or a 0.2 µm Immobilon-PSQ PVDF membrane (Merck Chemicals GmbH, Schwalbach, Germany) applying either by semi-dry method, using a PerfectBlue Semi-Dry-Electro Blotter (VWR International, Darmstadt, Germany) and Bjerrum buffer, or wet blot procedure PerfectBlue™ Tank Electro Blotter (VWR International) and Towbin buffer. Membranes were exposed to antibodies specific to IκBα (Santa Cruz Biotechnology, Heidelberg, Germany), IRAK-1 (New England Biolabs, Frankfurt am Main, Germany), p65 (Santa Cruz Biotechnology), α tubulin (Santa Cruz Biotechnology), lamin A/C (Santa Cruz Biotechnology), respectively, and subsequently incubated with corresponding HRP-conjugated secondary antibodies: anti-rabbit (New England Biolabs) and anti-mouse (Santa Cruz Biotechnology). Chemiluminescence was detected and documented by the ADVANCED Fluorescence and ECL Imager (Intas Science Imaging Instruments, Göttingen, Germany). If necessary, signal intensities were quantified by densitometric analysis using LabImage 1D software (Kapelan Bio-Imaging, Leipzig, Germany).

### RNA preparation, reverse transcription and quantitative RT-PCR (RT-qPCR)

For isolation of total RNA, a phenol-chloroform extraction using the Isol-RNA lysis reagent (5′ Prime, Hamburg, Germany) was carried out. Subsequent to reverse transcription with either TaqMan MicroRNA Reverse Transcription Kit or High-Capacity RNA-to-cDNA kit (Life Technologies) RT-qPCR was performed in a ViiA7 (Life Technologies). hsa-miR-146a-5p and RNU48 were quantified using TaqMan Fast Advanced Master Mix, while Fast SYBR Green Master Mix (Life Technologies) was used together with specific primer pairs (metabion GmbH, Planegg/Steinkirchen, Germany): IL-6 (sense: 5′-AATTCGGTACATCCTCGACGG-3′, anti-sense: 5′-TTGGAAGGTTCAGGTTGTTTTCT-3′), IL-8 (sense: 5′-ACTGAGAGTGATTGAGAGTGGAC-3′, anti-sense: 5′-AACCCTCTGCACCCAGTTTTC-3′), IRAK-1 (sense: 5′-TGAGGAACACGGTGTATGCTG-3′, anti-sense: 5′-GTTTGGGTGACGAAACCTGGA-3′), RPS18 (sense: 5′-GCGGCGGAAAATAGCCTTTG-3′, anti-sense: 5′-GATCACACGTTCCACCTCATC-3′). The fold-induction was calculated using the 2^−ΔΔct^ method^54^ and RT-qPCR results were normalized to the corresponding control cells.

### Statistics

The statistical data analysis was performed using the GraphPad Prism 6 (GraphPad, La Jolla, USA) software. Log-transformed data was used for statistical tests to allow treatment of data as gaussian. Two-tailed Student’s *t*-test or 2way ANOVA followed by Sidak’s multiple comparison test were employed as indicated. p-values ≤  0.05 were considered significant and depicted with one (p ≤ 0.05), two (p < 0.01), three (p < 0.001) or four (p < 0.0001) asterisks (*), hash marks (#) or paragraphs (§).

### Mathematical modelling

The mathematical model, which accounts for activation of the NF-κB signalling pathway by flagellin and IL-1β, was developed using ordinary differential equations (Supplementary Table S1). The model consisted of 10 variables, 20 kinetic reactions and 24 parameters (Supplementary Tables S2–S4). Four model parameters were characterized using data from literature, six are fixed at certain values (e.g. *flagellin* and *IL1β*), and the rest were estimated by fitting model simulations to 96 data points (Fig. 5b, further representative data sets can be found at https://tinyurl.com/LpneuSupp). The parameter estimation was performed using SBtoolbox 2 package in Matlab 2012b^55^. For parameter estimation, a combination of scatter search^56^ and downhill simplex^57^ methods were used to identify the optimal parameter set that minimizes the distance between model simulation and experimental data (Fig. 5b). For reproducibility, the model is available at https://goo.gl/ExPbpg via Biomodel database.

To evaluate identifiability of the estimated model parameters, we performed profile likelihood analysis^58^. Such an analysis allowed us to examine whether the estimated value for each unknown parameter was identifiable in the defined parameter interval and to determine confidence intervals for the estimated parameter values (see supplementary materials for details).

To validate the model with experimental data showing reduced *IL-8* expression of lung epithelial cells upon flagellin treatment after pre-stimulation with IL-1β (Figs 3d and 6A), the model was configured according to the experimental design (*IL1β* = 1 for [0 2] h and *flagellin* = 100 for [6 9] h). The simulated values of *mIL8* and *IRAK1*_*tot*_ (t = 9 h) were compared with the corresponding data in Figs 5c and 6b, respectively. Similarly, for comparison of simulation results with further experimental data demonstrating a reduced *IL-8* expression of *L*. *pneumophila* infected alveolar epithelial cells after co-culture with *L*. *pneumophila*-infected macrophages (Figs 3a and 6c), we set *IL1β* = 0.7 for [0 48] h and *flagellin* = 79.7 for [52 55] h. We extracted the simulated values of *mIL8* and *IRAK1*_*tot*_ at 55 h and are plotted the comparisons in Figs 5d and 6d. To imitate the knockdown of IRAK-1 by siRNA (Supplementary Fig. S11c), we set *IRAK1*(0) = 0.22 and ${k}_{IRAK1}^{syn}$ = 0.22 * 0.0961. We simulated the kinetic model and extracted the steady states of *mIL8* and *IRAK1*_*tot*_. The comparisons between experimental data and the simulations are plotted in Figs 6e and S11a.

To identify parameters with strong impact on the steady state (Supplementary Fig. S12) and the overall production (Fig. 5e) of IL-8 $(IL{8}_{tot\,}^{prod}\,=\,{\int }_{0}^{100}IL8{(t)}^{+}dt,\,IL8{(t)}^{+}\,=\,{k}_{IL8}^{transl}\,\cdot \,mIL8)$, we performed a sensitivity analysis calculating the difference of $IL{8}_{tot}^{prod}$ as a result of 10% change of the parameter’s nominal values. If the sensitivity index of a parameter is close to one or minus one, the 10% change of the parameter value can result in 10% increase or decrease of $IL{8}_{tot}^{prod}$, respectively. If the sensitivity index of a parameter is close to zero, the change of $IL{8}_{tot}^{prod}$ is negligible. To compare the influence of ${k}_{IRAK1p}^{deg}$ on IL-8 production with another parameter ${k}_{NFkB}^{loss}$, we perturbed their nominal values by 25% and the corresponding dynamics of the IL-8 production over time are plotted (Fig. 5e, inner graph).

To simulate the effect of *τ* and ${t}_{1/2}^{IRAK1p}\,$on the overall production of IL-8 $(IL{8}_{tot}^{prod}={\int }_{0}^{{\rm{\tau }}+{\rm{c}}}IL8{(t)}^{+}dt,$ $IL8{(t)}^{+}={k}_{IL8}^{transl}\cdot mIL8)$, Fig. 7A, we calculated the value $IL{8}_{tot}^{prod}$ using different combinations of the two parameters, which were sampled 100 times within the interval of [0 45] h and ${k}_{IRAK1p}^{deg}$ * [10^−1^ 10^1^], respectively. Different combinations of *τ* and ${t}_{1/2}^{IRAK1p}$ were selected to plot the corresponding dynamics of *IL8* and *IRAK1*_*tot*_ over time (Fig. 7B). The detailed description of the mathematical modelling can be found in the supplementary material.

## Electronic supplementary material

Supplementary Information
