# Supplementary material for: THP-1-derived macrophages render lung epithelial cells hypo-responsive to Legionella pneumophila – a systems biology study
Source: Sci Rep. 2017 Sep 20;7:11988. doi: 10.1038/s41598-017-12154-4 (PMC5607273; doi:10.1038/s41598-017-12154-4)
Supplement: Supplementary file 1 — Supplementary Information [file 41598_2017_12154_MOESM1_ESM.pdf]

THP-1-derived macrophages render lung epithelial cells hypo-responsive to Legionella pneumophila – a systems biology study

Christine Schulz[1], Xin Lai[2], Wilhelm Bertrams[1], Anna Lena Jung[1], Alexandra Sittka-Stark[1], Christina Elena Herkt[1], Harshavadhan Janga[3], Katja Zscheppang[4], Christina Stielow[1], Leon Schulte[3], Stefan Hippenstiel[4], Julio Vera[2], Bernd Schmeck[1,5*]

[1]Institute for Lung Research, Universities of Giessen and Marburg Lung Center, Philipps-University Marburg, Member of the German Center for Lung Research (DZL), Marburg, Germany.

[2]Laboratory of Systems Tumor Immunology, Department of Dermatology, Friedrich-Alexander-University of Erlangen-Nürnberg (FAU) and Universitätsklinikum Erlangen, Erlangen, Germany.

[3]Institute for Lung Research/iLung, Research Group "RNA-Biology of Inflammation & Infection", Philipps University, Marburg, Germany

[4]Department of Internal Medicine/Infectious Diseases and Respiratory Medicine, Charité – University Medicine Berlin, Germany

[5]Department of Medicine, Pulmonary and Critical Care Medicine, University Medical Center Giessen and Marburg, Philipps-University Marburg, Member of the German Center for Lung Research (DZL), Marburg, Germany.

* corresponding author:

E-Mail: bernd.schmeck@uni-marburg.de (BS)

Supplementary information

Primary cell preparation

Alveolar epithelial type 2 cells

Fresh lung explants were obtained from 3 patients suffering from bronchial carcinoma, who underwent lung resection at local thoracic surgeries. The study was approved by the ethics committee at the Charité clinic (projects EA2/050/08 and EA2/023/07) and at Philipps University Marburg (project 224/12) and written informed consent was obtained from all patients. Alveolar epithelial type 2 cells (AECII) were isolated as described before[1]. Briefly, human lung tissue was cleared from alveolar macrophages (AMs) by repeated perfusion, finely minced and digested with trypsin type I (0.25%) and DNAse (350U/ml). The digested tissue was filtered through gauze strainers and for differential adherence of residual AM cells were incubated in HBSS:Hybridoma-SFM medium. The non-adherent cell fraction was collected and layered on a Pancoll discontinuous gradient. After centrifugation, AECII at the interfacial layer were washed and finally seeded at $5*10^5$ cells/well. AECII cells were cultured for 6 days in SABM medium with SAGM BulletKit supplements (Lonza) before experimentation.

Blood-derived macrophages

Blood-derived human macrophages were obtained as described before[2]. Briefly, monocytes were isolated from buffy coats obtained from the center for transfusion medicine and hemotherapy, University Hospital Giessen and Marburg GmbH by Miltenyi MACS CD14 positive selection according to the manufacturer´s instructions. After 6 days of culture in

RPMI 1640 (1 % glutamine and 1 % Human Serum Type AB), experiments were performed. All donors gave informed written consent.

Lactat dehydrogenase (LDH) release - assay

LDH-release in the supernatants of infected (MOI 0.5 at 48 h time point) primary human alveoar macrophages and THP-1 cells was analyzed using the cytotoxicity detection kit from Roche Diagnostics GmbH according to the supplier's protocol (Fig S14).

Model description

Based on the scheme (Fig. 5a), which summarizes the most relevant molecular interactions in alveolar epithelial cells during *L. pneumophila* infection, we developed a model of ordinary differential equations to describe the NF-κB signaling pathway activated by flagellin and IL-1β [3]. In lung epithelial cells, flagellin and IL-1β can activate the pathway through TLR5 and IL-1 receptor, respectively. After recognizing these ligands, recruitment of protein effectors such as IRAK-4 can result in the phosphorylation of IRAK-1. Phosphorylated IRAK-1 leads to the activation of IKK, whose active form can release free NF-κB by degrading IκBα. The transcription factor NF-κB enters the nucleus and upregulates the transcription of many genes. Among these genes the model includes *IκBα* and *IL-8* mRNA, which are further translated into IκBα and IL-8, respectively. As IL-8 is a pro-inflammatory cytokine, it was used as a surrogate for the inflammatory response triggered by lung epithelial cells during *L. pneumophila* infection. The developed model consisted of 10 variables and 20 kinetic reactions (S1-S3 Tables).

Parameter estimation

The four time-series experiments used for parameter estimation included sustained stimulation of lung epithelial cells (A549) by different amounts of *L. pneumophila* (MOI 10 and 100) and sustained or temporal stimulation of A549 cells by IL-1β (1 ng/mL). To make the two stimuli comparable, we assumed that flagellin is the dominate stimuli during *L. pneumophila* infection of alveolar epithelial cells. This was in line with previous experimental results demonstrating an unresponsiveness of A549 cells to TLR2 stimulation with *L. pneumophila* lipopolysaccharides and Pam3Cys (data not shown). Then, we converted the MOI 10 and MOI 100 of *L. pneumophila* into flagellin concentrations using the following calculation.

L. pneumophila has a single polar flagellum and each flagellum consists of ~$2*10^4$ copies of flagellin with a mass of ~48 kDa [4]. This results in a total amount of flagellin of ~$9.6*10^8$ Da for each bacterium. Since 1 Da equals ~$1.66*10^{-15}$ ng, each bacterial flagellum has ~$1.594*10^{-6}$ ng flagellin. $5*10^5$ lung epithelial cells were infected with either $5*10^6$ (MOI 10) or $5*10^7$ (MOI 100) bacteria in a volume of 1 mL. So, MOI 10 and MOI 100 correspond to flagellin concentrations of $1.594*10^{-6}*5*10^6=7.97$ ng/mL and $1.594*10^{-6}*5*10^7=79.7$ ng/mL, respectively. These numbers were used for fixing the initial values of the variable *flagellin* during the parameter estimation (i.e., MOI 10: *flagellin*(*0*)=7.97; MOI 100: *flagellin*(*0*)=79.7). Similarly, for the sustained stimulation with IL-1β the initial value of the variable *IL1β* was fixed at one (i.e., *IL1β*(*t*)=1) due to the reason that 1 ng/mL of IL-1β was used to stimulate the alveolar epithelial cells, and for the temporal stimulation its value was set to zero after 2 h (i.e., *IL1β*(0)=1 and *IL1β*(*t*>2)=0) because the medium was exchanged at that time point.

Among the 24 model parameters, four were characterized using biological knowledge and six were fixed. Specifically, the half-life ($t_{1/2}$) of molecular spices derived from data or literature were used to characterize the corresponding degradation rate constants using the equation $k_{var}^{deg} = \frac{log2}{t_{1/2}}$; we set $k_{IRAK1}^{syn} = k_{IRAK1}^{deg}$ and $k_{IKK}^{syn} = k_{IKK}^{deg}$ to make the steady states of *IRAK1* and *IKK* equal to their initial values when the inputs (i.e. *IL1β* and *flagellin*) of the system were zero; we set the values of K_1, K_2, K_3 and N_{tot} to one for simplicity. The remaining 14 parameters were estimated by fitting model simulations to the experimental data accounting for the temporal dynamics of NF-κB signaling pathways of lung epithelial cells in response to flagellin and IL-1β stimulation derived from our own experimental data (Fig. 5b). Of note for $IRAK1_{tot}$, since the experimental data showed a quite stable IRAK-1 protein expression until 12 h, we assumed that the stability of IRAK-1 protein is not affected upon infection of alveolar epithelial cells with a low amount (MOI 10) of *L. pneumophila*. The two stimuli (flagellin and IL-1β) were set as constants for sustained treatment of alveolar epithelial cells, whereas IL-1β was modeled as an impulse at time zero for 2 h using the step function for temporary challenge or co-culture experiments. Based on the knowledge about molecular reaction rates and signal transduction speeds within cells [5], the searching interval for unknown parameters was [$4*10^{-4}$ $2.5*10^{3}$]. This restricted the speed of biochemical reactions on a scale from seconds to hours. The searching interval of known parameters was constrained on their initial values with 10% variability (e.g. k_{IRAK1p}^{deg}, k_{IRAK1}^{deg} and k_{mIL8}^{deg}). To ensure a comprehensive search of the defined log transformed parameter space, we generated 5000 initial parameter sets using

the Latin Hypercube sampling method. Then, we used these parameter sets as inputs for parameter estimation which aims to minimize the cost function

$$\chi^2(p) = \sum_{i=1}^{M} \sum_{j=1}^{H} \left(\frac{y_i^{data}(t_j) - y_i(p, t_j)}{\sigma_{ij}^{data}} \right)^2,$$

where $y_i^{data}(t_j)$ denotes the measurement of the observable i at time point t_j. σ_{ij}^{data} represents the corresponding standard deviation of the experimental data, and $y_i(p, t_j)$ is the ith observable predicted by the model, which was configured with the parameter set p for time point t_j. M accounts for the number of observables and H for the number of time points measured. The optimal parameter set was characterized by a minimal distance between model simulations and experimental data and their values can be found in S4 Table. The parameter estimation was implemented using SBtoolbox2 package under Matlab2012b [6].

Parameter identifiability analysis

We adopted the profile likelihood method to generate a cost function profile for each estimated parameter [7]. Briefly, we used the optimal parameter set obtained from the parameter estimation step as a starting point. Then, for each estimated parameter we perturbed its value in the defined interval and re-optimised the other parameters to calculate the cost function profile over the defined parameter searching interval. Finally, we set the threshold $\Delta_\alpha = \chi^2(\alpha, df)$ with α=0.68 and df=1, which is the α quantile of the χ^2 (chi-square)-distribution with df degrees of freedom, to locate the confidence interval for each of the estimated parameters. The initial results showed that eight out of 14 unknown parameters are practically non-identifiable in the defined searching interval. Further

analyses showed that this issue was a result of strong correlations among some parameters. The parameters $k_{mIkB\alpha}^{transc}$, k_{NFkB}^{gain} and $k_{mIL8}^{transc2}$ showed not only strong correlation with each other but also high variances for the best 200 fittings out of the 5000 estimations (S13 Fig.). To resolve this issue, we fixed $k_{mIkB\alpha}^{transc}$ at its optimal value and re-performed parameter estimations using another 5000 parameter combinations and repeated the parameter identifiability analysis. The results showed that the other two parameters (k_{NFkB}^{gain} and $k_{mIL8}^{transc2}$) had much less variances after fixing the value of $k_{mIkB\alpha}^{transc}$ (S13 Fig.). Finally, eight out of 14 estimated parameters were identifiable and the others were practically non-identifiable (S8 Fig.).

Model validation

After calibration, the model was first validated with our data showing a hypo-responsiveness of lung epithelial cells to flagellin stimulation mediated by an initial challenge with IL-1β (Fig. 3d and 6a). Accordingly, the model was configured based on experimental procedure: *IL1β* was set to one for the first 2 h; after 4 h of incubation *flagellin* was set to 100; the simulated values of *mIL8* and $IRAK1_{tot}$ at the time point 9 h were extracted and compared with corresponding experimental data. These data originated from individual IL-1β or flagellin treatments as well as re-stimulations and untreated alveolar epithelial cells served as control. Both simulation results and experimental data were normalized to the maximum value of the four plotting scenarios (Fig. 5c and 6b).

The model was further validated by experimental data showing a hypo-responsiveness of lung epithelial cells to *L. pneumophila* infection upon co-culture with *L. pneumophila*-

infected macrophages (Fig. 3a and 6c). Accordingly, the model was configured based on the experimental procedure: *IL1β* was set to 0.7 in the interval of [0 48] h, as the measured concentration of IL-1β was 0.7 ng/mL after infection of macrophages with *L. pneumophila* (MOI 0.5) for 48 h (Fig. 1b); after 4 h of incubation, *flagellin* was set to 79.7; the simulated values of *mIL8* and $IRAK1_{tot}$ were extracted at the time point 55 h and compared with corresponding experimental data. For this purpose, alveolar epithelial cells were co-cultured with *L. pneumophila* infected macrophages (MOI 0.5), directly infected with *L. pneumophila* (MOI 100) or both. Data from untreated pulmonary epithelial cells was used as control. Both, simulation results and experimental data were normalized to the maximum value of the four scenarios for plotting (Fig. 5d and 6d).

In IRAK-1 knockdown experiments, IRAK-1 protein expression was reduced by 78% (S11c Fig.). To imitate the knockdown of IRAK-1 protein as a result of siRNA transfection, we set *IRAK1*(0)=0.22 and k_{IRAK1}^{syn}=0.22*0.0961. We simulated the kinetic model and extracted the steady states of *mIL8* and $IRAK1_{tot}$. For comparison of model simulations and experimental data, we normalized measured and simulated expressions of $IRAK1_{tot}$ to the control scenario and *mIL8* to its maximum value in the individual scenarios (Fig. 6e).

Sensitivity analysis

To identify parameters' impact on the steady states of model variables, we computed their sensitivity indexes using the following equation

$$S_{ij} = \frac{(x_i^{ss}(p_j + \Delta p_j) - x_i^{ss}(p_j))/x_i^{ss}(p_j)}{\Delta p_j/p_j},$$

where $x_i^{ss}(p_j)$ denotes the nominal steady state of the model variable x_i when the parameter p_j is fixed at its nominal value. $x_i^{ss}(p_j + \Delta p_j)$ represents a new steady state after increasing the parameter value by 10%. The calculated S_{ij} denotes how much the change of the steady state of x_i in percent (normalized by $x_i^{ss}(p_j)$) is caused by the change of the corresponding parameter p_j in percentage (normalized by p_j). If S_{ij} is greater than zero, the change of p_j leads to the change of x_i^{ss} in the same direction (i.e. decrease or increase), otherwise the change of p_j leads to the opposite change of x_i^{ss}. The bigger the distance value of S_{ij} to zero is, the more sensitive is x_i^{ss} to p_j (S12 Fig.). Similarly, instead of using the steady states of model variables, we calculated the sensitivity indexes for model parameters by means of the overall production of IL-8 ($IL8_{tot}^{prod}$) during a certain time period, which was defined by the following equation

$$IL8_{tot}^{prod} = \int_0^{100} IL8(t)^+ \, dt, \quad IL8(t)^+ = k_{IL8}^{transl} \cdot mIL8,$$

where the time period was set to 100 h, at which *IL8* already reaches its steady state. To imitate the experimental condition, we set *IL1β*=0.001 up to 48 h and *flagellin*=1 from 52 h. The corresponding sensitivity indexes were calculated using the following equation

$$S_{ij} = \frac{(IL8_{tot}^{prod}(p_j + \Delta p_j) - IL8_{tot}^{prod}(p_j))/IL8_{tot}^{prod}(p_j)}{\Delta p_j / p_j}.$$

Besides, we perturbed the nominal values of k_{IRAK1p}^{deg} and k_{NFkB}^{loss} by 25% to show their impact on the temporal dynamics of $IL8_{tot}^{prod}$. The results are shown in Fig. 5e.

The *in silico* experiment

We used the following equation to calculate the overall production of IL-8 during a certain time period (Fig. 7a)

$$IL8_{tot}^{prod} = \int_0^{\tau+c} IL8(t)^+ \, dt, \quad IL8(t)^+ = k_{IL8}^{transl} \cdot mIL8,$$

where the time period starts from zero and ends at the time point, which is *c* h after the beginning (τ) of flagellin stimulation. We set *c* to 30 h, as simulations showed that after this time period *IL8* reaches its steady states after A549 cells stimulation with IL-1β alone for 2 h. $IL8_{tot}^{prod}$ was calculated for different combinations of τ and $t_{1/2}^{IRAK1p}$. τ and $t_{1/2}^{IRAK1p}$ values were sampled 100 times in the interval of [0 45] h and k_{IRAK1p}^{deg}*[10^{-1} 10^{1}], respectively. Given individual simulations, we used different values of τ (Fig. 7b-h), $t_{1/2}^{IRAK1p}$ (Fig. 7b-v) or both parameters (Fig. 7b-d) and plotted the corresponding temporal dynamics of *IL8* and $IRAK1_{tot}$.

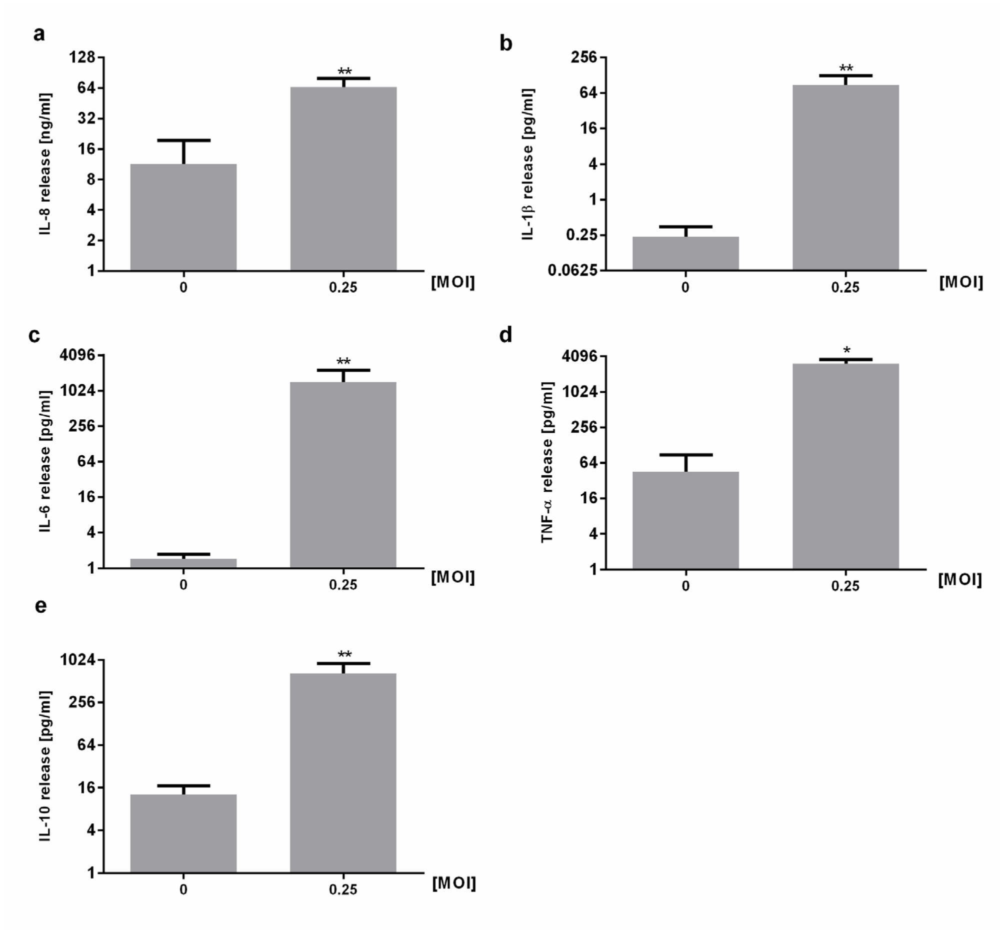

Figure S1: Cytokine release of blood-derived macrophages upon infection with *L. pneumophila*. Blood-derived macrophages (BDM) were stimulated with *L. pneumophila* (MOI 0.25) for 48 h. (A) IL-8 secretion was measured by ELISA, (B-E) secretion of IL-1β, TNF-α, IL-6 and IL-10 were measured using Multiplex Luminex Assay. Data are shown as mean ± SEM (n = 3). Student´s t-test was performed as described: * $p \leq 0.05$, ** $p \leq 0.01$.

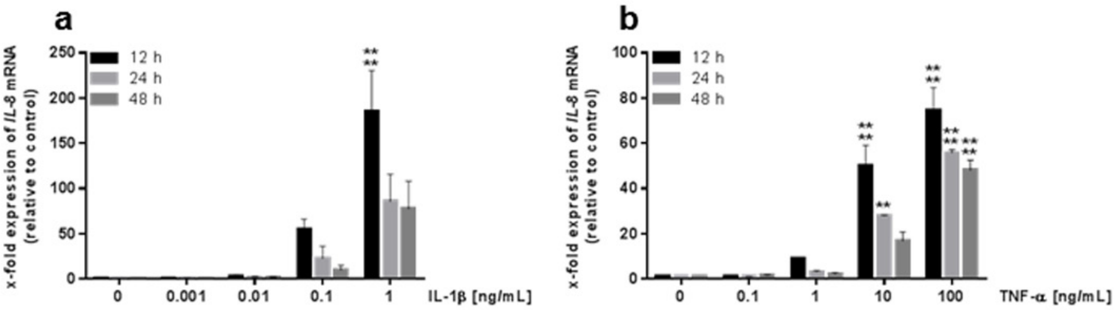

S2 Fig. Susceptibility of A549 cells to IL-1β or TNF-α treatment. A549 cells were incubated with increasing concentrations of either IL-β (A) or TNF-α (B) for 12, 24 and 48 h, respectively. *IL-8* expression was analyzed by RT-qPCR and normalized to the corresponding unstimulated control. Data are shown as mean ± SEM (n = 3). Statistical analysis was performed as described: * compared to matching control; ** $p \leq 0.01$, **** $p \leq 0.0001$.

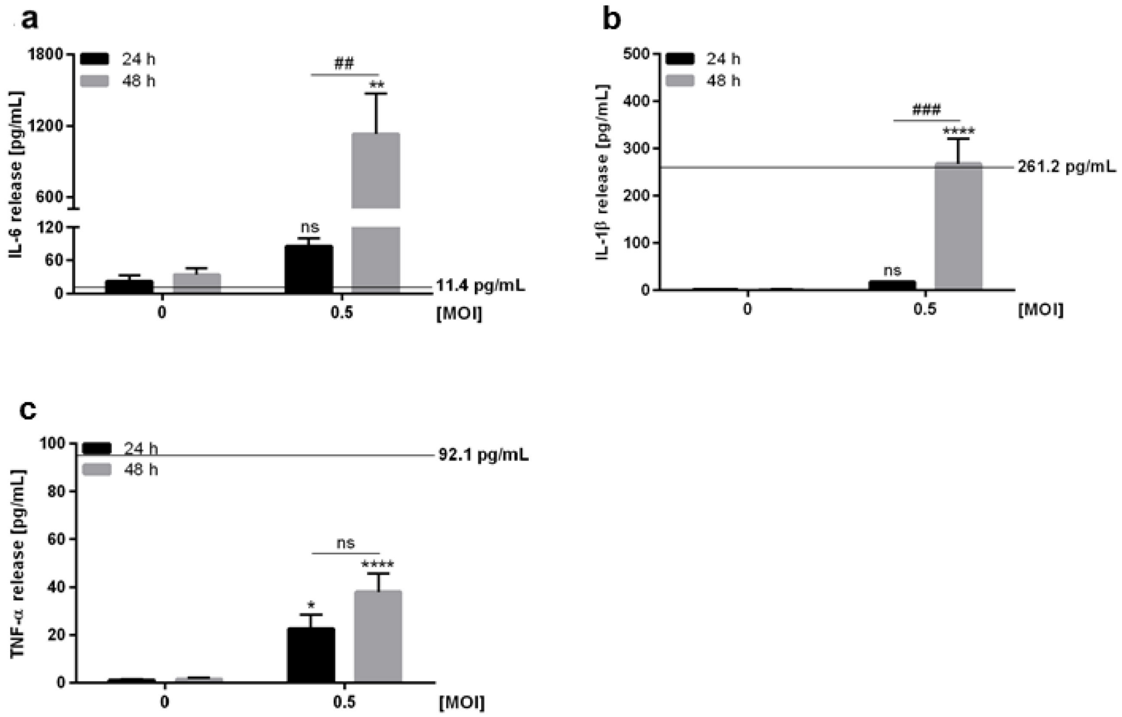

S3 Fig. Pro-inflammatory cytokine release in a co-culture system of *L. pneumophila*-infected THP-1 and A549 cells. THP-1 cells were stimulated with *L. pneumophila* (MOI 0.5, 24 or 48 h). IL-6 (A), IL-1β (B), and TNF-α (C) secretion of co-cultures was measured by ELISA; black lines: cytokine release of THP-1 cells 48 h post infection calculated according to figure 1. Statistical analysis was performed as described: * compared to corresponding control, # compared to equally treated 24 h sample; * $p \leq 0.05$, ** or ## $p \leq 0.01$, ### $p \leq 0.001$, **** $p \leq 0.0001$, ns = not significant.

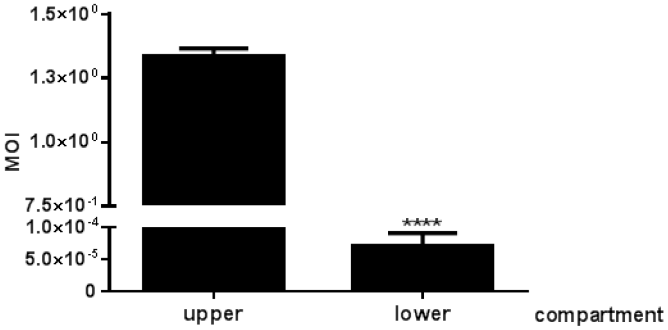

S4 Fig. *L. pneumophila* transfer to the lower compartment in a co-culture system of THP-1 and A549 cells. THP-1 cells (upper compartment) co-cultured with A549 cells (lower compartment) were infected with *L. pneumophila* (MOI 1, 48 h). An input control and the supernatants of the lower compartment were plated on BCYE agar. CFUs of the lower compartment were counted after three days compared to input controls. MOIs were calculated by means of counted CFUs and the following cell numbers: $8*10^4$ cells/cm^2 (THP-1 cells) and $1.2*10^5$ cells/cm^2 (A549 cells). Data are shown as mean ± SEM (n = 3). Statistical analysis: two-tailed Student´s t-test; * compared to the upper compartment; **** $p \leq 0.0001$.

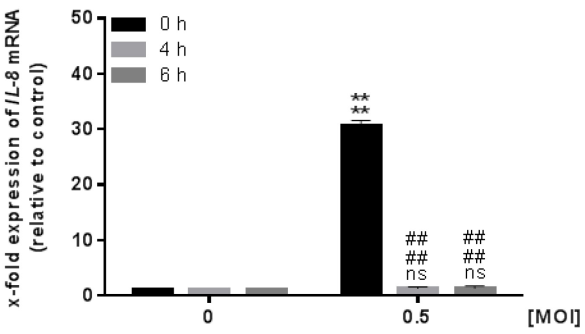

S5 Fig. Removal of *L. pneumophila*-infected THP-1 cells leads to basal *IL-8* expression in co-cultured A549 cells. THP-1 cells were stimulated with *L. pneumophila* (MOI 0.5) for 48 h. Upon their removal, A549 cells were incubated in fresh medium for 4 or 6 h, respectively. *IL-8* expression was analyzed by RT-qPCR and normalized to the corresponding untreated control. Data are shown as mean ± SEM (n = 4). Statistical analysis was performed as described: * compared to matching control, # compared to corresponding *L. pneumophila*-infected sample; **** or #### p ≤ 0.000.1, ns = not significant.

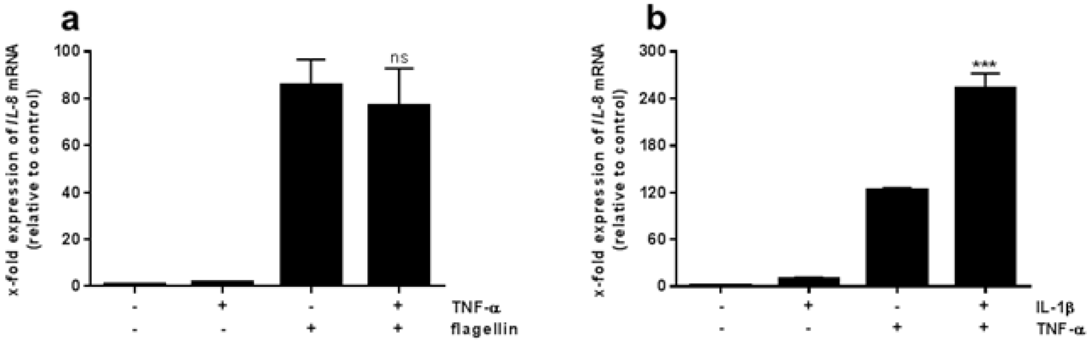

S6 Fig. TNF-α is insufficient to induce the hypo-responsiveness of epithelial cells. A549 cells were stimulated with (A) TNF-α (100 ng/mL) or (B) IL-1β (1 ng/mL) for 2 h. Following medium renewal and incubation for 4 h, A549 cells were stimulated with (a) flagellin (100 ng/mL) or (b) TNF-α (100 ng/mL) for 3 h. *IL-8* expression was analyzed by RT-qPCR and normalized to the unstimulated control. Data are shown as mean ± SEM (n = 4). Statistical analysis: two-tailed Student´s t-test; * compared to single TNF-α treatment; *** $p \leq 0.001$, ns = not significant.

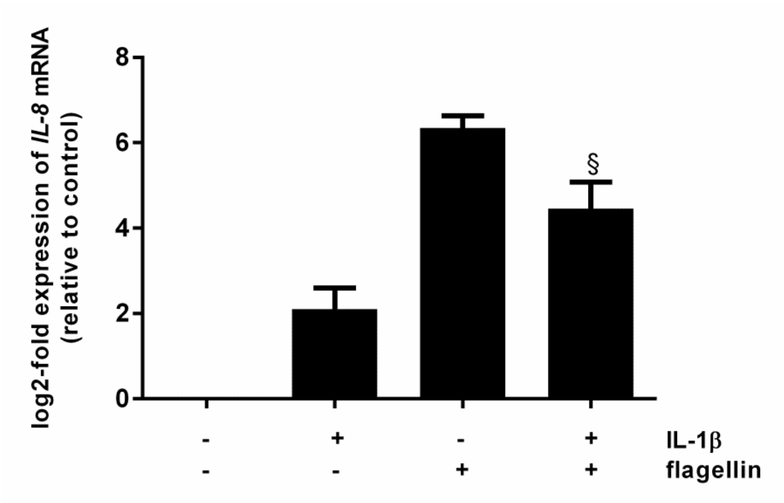

S7 Fig Paracrine mechanisms render alveolar epithelial type II cells hypo-responsive to *L. pneumophila* infection. AECII cells were stimulated with IL-1β (1 ng/mL) for 2 h. Following medium renewal (4 h incubation), cells were stimulated with flagellin (100 ng/mL) for 3 h. *IL-8* expression was analysed by RT-qPCR. Data are shown as mean ± SEM (n = 3). Two-tailed Student´s t-test was performed as described: § compared to flagellin stimulation; § p ≤ 0.0.5.

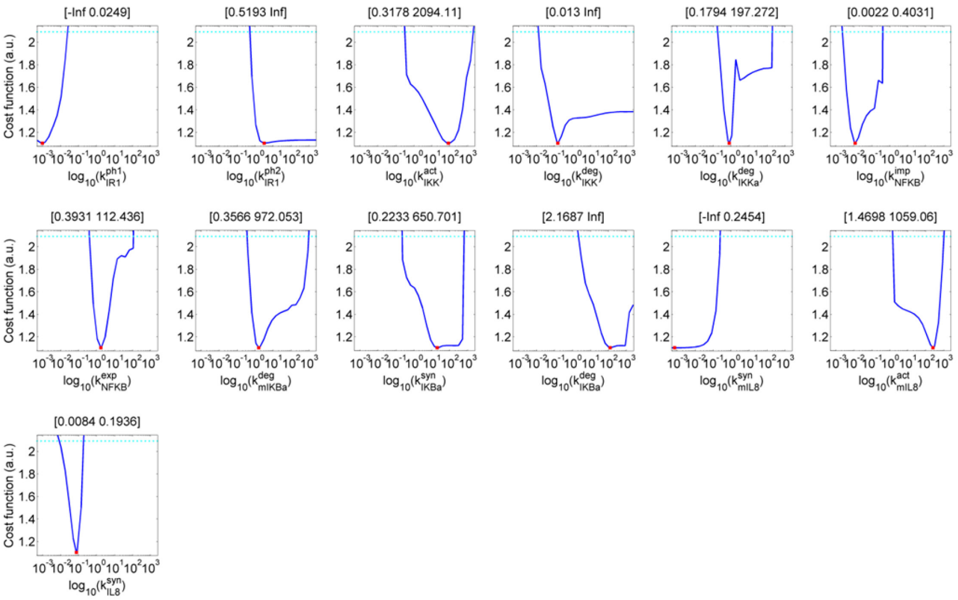

S8 Fig. Cost function likelihood profiles for the estimated parameters. The parameters were perturbed in the defined interval. The optimal parameter values are indicated by red squares. The cyan dotted line is the threshold that indicates a 68% confidence level. The points of profile and threshold pass-over determined likelihood-based confidence intervals (sub-figure titles). Cost function likelihood profiles that have a unique minimum but can cross the confidence threshold in one direction revealed practically non-identifiable parameters (e.g. k_{IRAK1}^{ph1}, k_{IRAK1}^{ph2}, k_{IKK}^{deg}, k_{IkBa}^{loss}, and $k_{mIL8}^{transc1}$). The remaining parameters are identifiable and have finite confidence intervals. Discontinuities in the profile likelihood stem from local minima that govern the profile likelihood in remote regions.

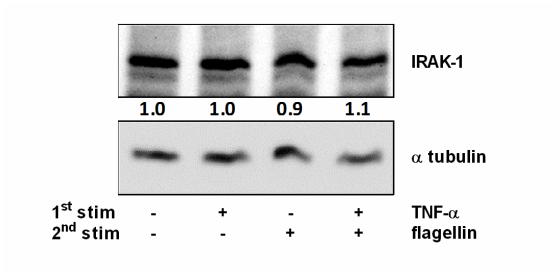

S9 Fig. IRAK-1 protein levels are not affected by TNF-α treatment. A549 cells were stimulated with TNF-α (100 ng/mL, 2 h). After medium renewal and incubation for 4 h, A549 cells were stimulated with flagellin (100 ng/mL, 3 h). Protein levels of IRAK-1 and α tubulin were analyzed by western blotting and quantified. One out of four representative blots is shown.

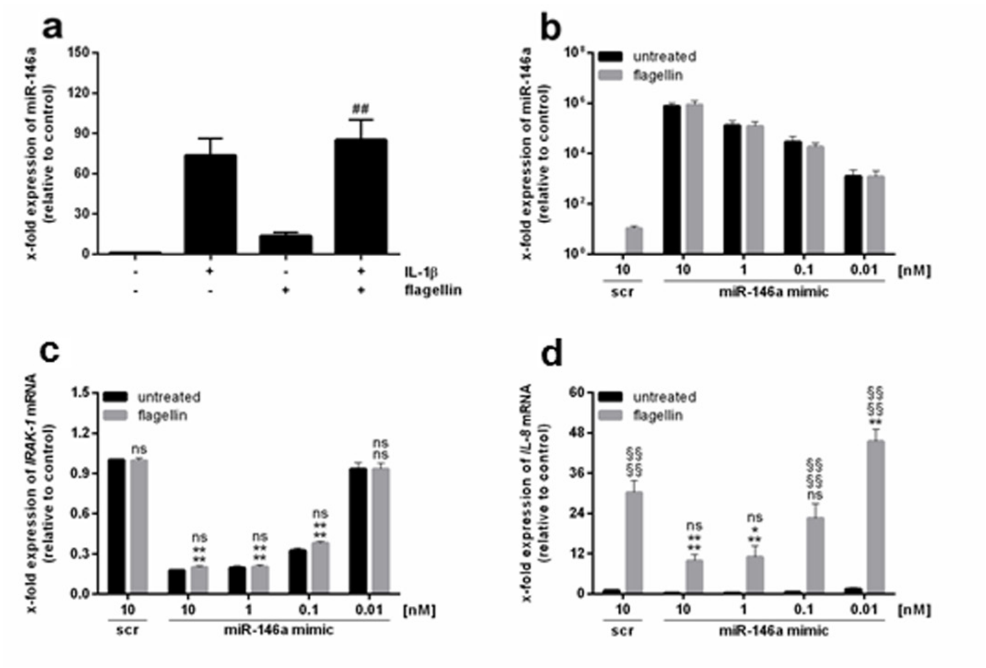

S10 Fig. Hypo-responsiveness cannot be mimicked by miR-146a overexpression. (a) A549 cells were stimulated with IL-1β (1 ng/mL, 2 h), followed by medium renewal and incubation for 4 h. Next, A549 cells were stimulated with flagellin (100 ng/mL, 3 h). miR-146a expression was analyzed by RT-qPCR (mean ± SEM, n = 4). (b-d) Cells were transfected with control (scr) or miR-146a mimic (24 h) prior to flagellin stimulation (100 ng/mL, 3 h). (b) miR-146a, (c) *IRAK-1*, and (d) *IL-8* expression was analyzed by RT-qPCR and normalized to an untreated scr (mean ± SEM, n = 4). Statistical analysis: (a) two-tailed Student´s t-test; # compared to flagellin treatment; ## $p \leq 0.01$; (c, d) as described: * compared to corresponding scr, § compared to corresponding untreated sample; ** $p \leq 0.01$, *** $p \leq 0.001$, **** or §§§§ $p \leq 0.0001$; ns = not significant.

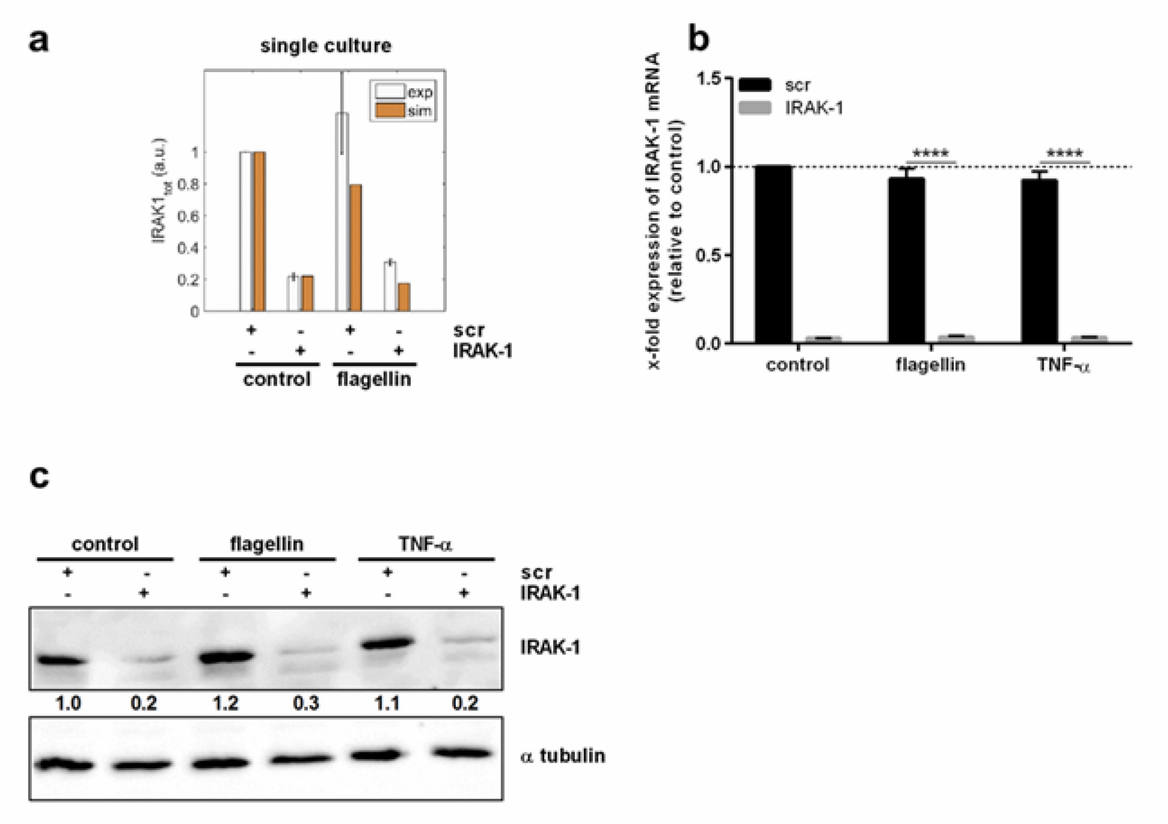

S11 Fig. Knockdown of IRAK-1 in A549 cells. A549 cells were transfected with control (scr) or IRAK-1 siRNA. Upon 48 h, cells were stimulated with either flagellin (100 ng/mL) or TNF-α (100 ng/mL) for 3 h. (a) The model was used to predict IRAK-1 expression upon its depletion, which was in accordance with the experimental data. IRAK-1 (b) mRNA and (c) protein expression was analyzed by RT-qPCR and western blotting followed by quantification, respectively. (b) mRNA expression was normalized to an untreated control. Data are shown as mean ± SEM (n = 4). (c) One out of four representative blots is shown.

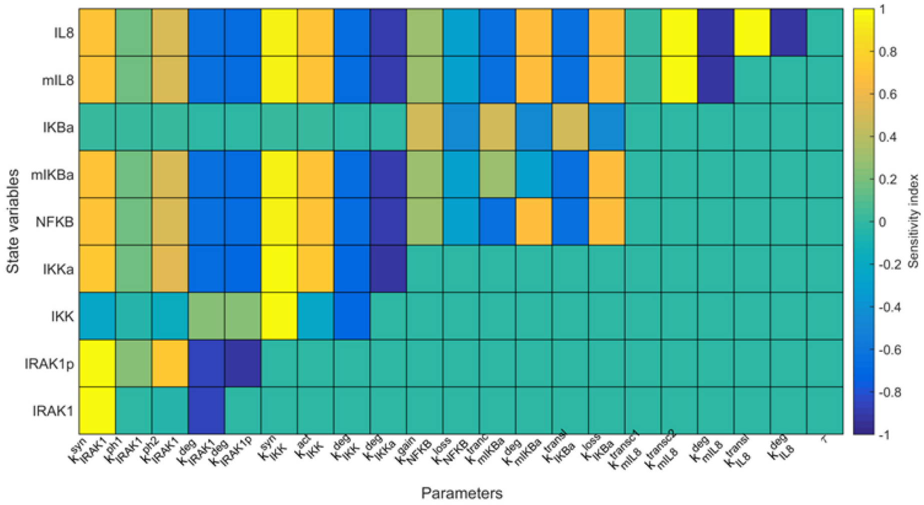

S12 Fig. Sensitivity analysis of model parameters against the steady states of model variables. The x and y axis denote model parameters and variables, respectively. The color represents the values of sensitivity indexes S_{ij}. The more yellow (S_{ij}>0) or blue (S_{ij}<0) the color is, the more sensitive is the steady state of a model variable to a parameter (i.e. small change of the parameter value can lead to a significant change in the steady state of the model variable). The green color (i.e. S_{ij}=0) indicates that the steady state of a model variable is insensitive to a parameter (i.e. change of the parameter value cannot alter the steady state of the model variable).

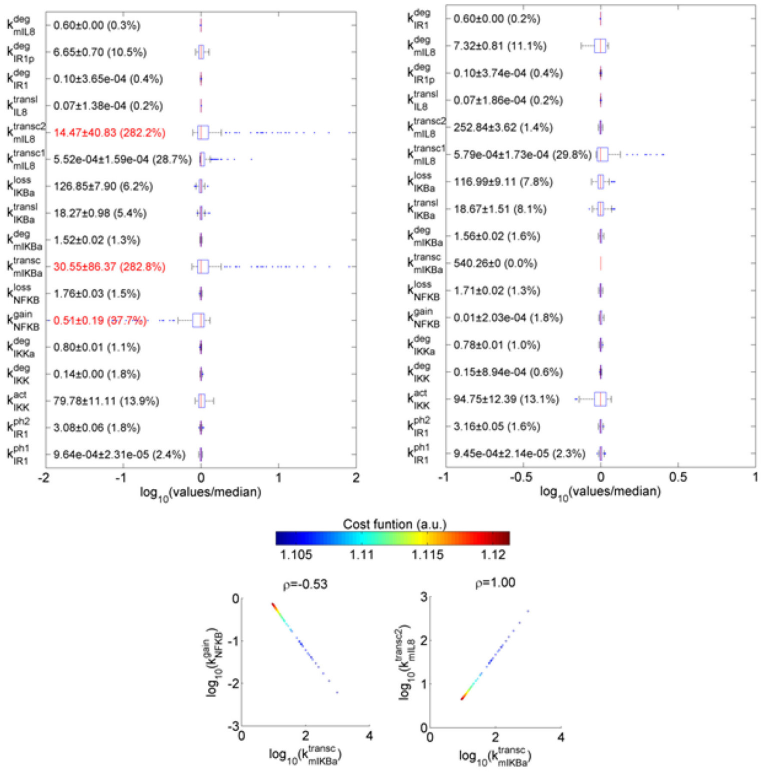

S13 Fig. Parameter uncertainty and correlation analyses. Distribution of the estimated parameter values (mean ± SD) for the best 200 of 5000 fits before (upper left panel) and after (upper right panel) fixing the value of $k_{mIkB\alpha}^{transc}$, which was a practically unidentifiable parameter and showed strong correlation with k_{NFkB}^{gain} and $k_{mIL8}^{transc2}$ (lower panel). Before fixing the value of $k_{mIkB\alpha}^{transc}$, three parameters (highlighted in red) had large variances (SD/mean*100%). After fixing $k_{mIkB\alpha}^{transc}$ at its optimal value, k_{NFkB}^{gain} and $k_{mIL8}^{transc2}$ showed much reduced variances.

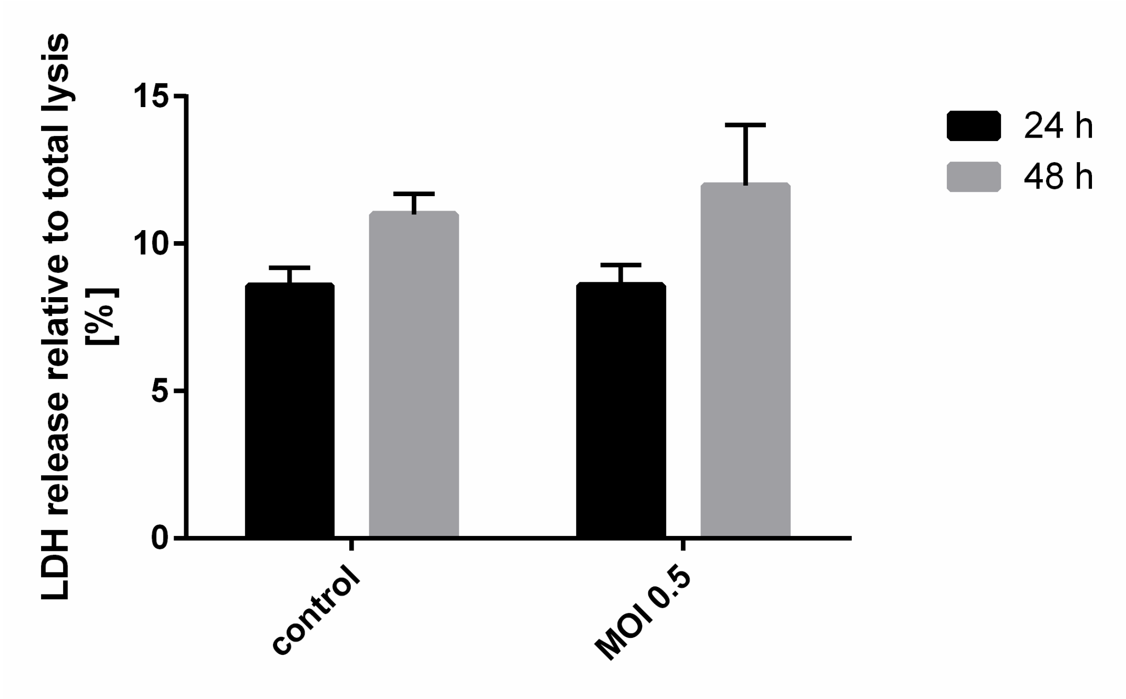

Figure S14: LDH release of THP-1 cells infected with *Legionella pneumophila*. 24 h/48 h after infection with *L pneumophila*, LDH was measured in the supernatant of THP-1 cells as an indicator of cytotoxicity. Data are shown as mean percentage ± SEM (n = 4) of total lysis.

Nr.	Model equation
(1)	$\frac{d(IRAK1)}{dt} = k_{IRAK1}^{syn} - k_{IRAK1}^{deg} \cdot IRAK1 - k_{IRAK1}^{ph1} \cdot IRAK1 \cdot flagellin - k_{IRAK1}^{ph2} \cdot IRAK1 \cdot IL1\beta$
(2)	$\frac{d(IRAK1p)}{dt} = k_{IRAK1}^{ph1} \cdot IRAK1 \cdot flagellin + k_{IRAK1}^{ph2} \cdot IRAK1 \cdot IL1\beta - k_{IRAK1p}^{deg} \cdot IRAK1p$
(3)	$\frac{d(IKK)}{dt} = k_{IKK}^{syn} - k_{IKK}^{act} \cdot IKK \cdot IRAK1p - k_{IKK}^{deg} \cdot IKK$
(4)	$\frac{d(IKKa)}{dt} = k_{IKK}^{act} \cdot IKK \cdot IRAK1p - k_{IKKa}^{deg} \cdot IKKa$
(5)	$\frac{d(NFkB)}{dt} = k_{NFkB}^{gain} \cdot IKKa \cdot \frac{N_{tot} - NFkB}{K_1 + IkB\alpha + IKKa} - k_{NFkB}^{loss} \cdot \frac{IkB\alpha \cdot NFkB}{K_2 + NFkB}$
(6)	$\frac{d(mIkB\alpha)}{dt} = k_{mIkB\alpha}^{transc} \cdot NFkB - k_{mIkB\alpha}^{deg} \cdot mIkB\alpha$
(7)	$\frac{d(IkB\alpha)}{dt} = k_{IkB\alpha}^{transl} \cdot mIkB\alpha - k_{IkB\alpha}^{loss} \cdot IKKa \cdot IkB\alpha \cdot \frac{(N_{tot} - NFkB)}{K_3 + IkB\alpha + IKKa}$
(8)	$\frac{d(mIL8)}{dt} = k_{mIL8}^{transc1} + k_{mIL8}^{transc2} \cdot NFkB - k_{mIL8}^{deg} \cdot mIL8$
(9)	$\frac{d(IL8)}{dt} = k_{IL8}^{transl} \cdot mIL8 - k_{IL8}^{deg} \cdot IL8$
(10)	$IRAK1_{tot} = IRAK1 + IRAK1$

S1 Table. Model equations. The model equations are derived using mass action kinetics, except for the variables: *mIκBα*, *IκBα* and *NFκB*. The corresponding equations for those three variables were simplified from a comprehensive model that considered all the complexes formed by IKK, IκBα and NF-κB and their interactions[8].

Reaction	Kinetics	Remark
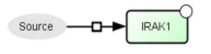	k_{IRAK1}^{syn}	Synthesis of IRAK-1
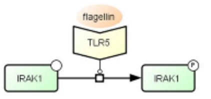	$k_{IRAK1}^{ph1} \cdot IRAK1 \cdot flagellin$	Phosphorylation of IRAK-1 by flagellin through TLR5
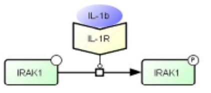	$k_{IRAK1}^{ph2} \cdot IRAK1 \cdot IL1\beta$	Phosphorylation of IRAK-1 by IL-1β through IL1-R
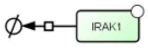	$k_{IRAK1}^{deg} \cdot IRAK1$	Degradation of IRAK-1
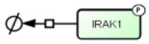	$k_{IRAK1p}^{deg} \cdot IRAK1p$	Degradation of phosphorylated IRAK-1
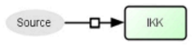	k_{IKK1}^{syn}	Synthesis of IKK
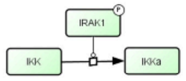	$k_{IKK}^{act} \cdot IKK \cdot IRAK1p$	Activation of IKK by phosphorylated IRAK-1
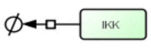	$k_{IKK}^{deg} \cdot IKK$	Degradation of IKK
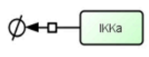	$k_{IKKa}^{deg} \cdot IKKa$	Degradation of active IKK
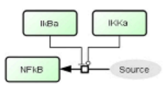	$k_{NFkB}^{gain} \cdot IKKa \cdot \frac{N_{tot} - NFkB}{K_1 + IkB\alpha + IKKa}$	Gain rate of free NF-κB mediated by IκBα and IKKα
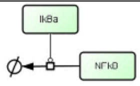	$k_{NFkB}^{loss} \cdot \frac{IkB\alpha \cdot NFkB}{K_2 + NFkB}$	Loss of free NF-κB mediated by IκBα
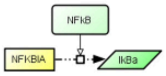	$k_{mIkB\alpha}^{transc} \cdot NFkB$	Transcription of *IκBα* mRNA mediated by transcriptional active NF-κB
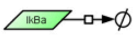	$k_{mIkB\alpha}^{deg} \cdot mIkB\alpha$	Degradation of *IκBα* mRNA
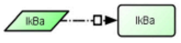	$k_{IkB\alpha}^{transl} \cdot mIkB\alpha$	Translational of IκBα from its mRNA
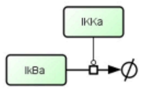	$k_{IkB\alpha}^{loss} \cdot IKKa \cdot IkBa \cdot \frac{(N_{tot} - NFkB)}{K_3 + IkB\alpha + IKKa}$	Loss of free IκBα mediated by IKKα and NF-κB
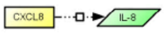	$k_{mIL8}^{transc1}$	Basal transcription of *IL-8* mRNA
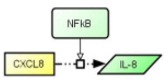	$k_{mIL8}^{transc2} \cdot NFkB$	Transcription of *IL-8* mRNA mediated by transcriptional active NF-κB
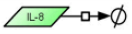	$k_{mIL8}^{deg} \cdot mIL8$	Degradation of *IL-8* mRNA
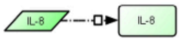	$k_{IL8}^{transl} \cdot mIL8$	Translational of IL-8 from its mRNA
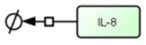	$k_{IL8}^{deg} \cdot IL8$	Degradation of IL-8

S2 Table. Description of the reaction kinetics used in the model for each interaction considered.

Model variable	Description	Initial condition (a.u.)*
IRAK-1	Non-phosphorylated IRAK-1	1
IRAK-1p	Phosphorylated IRAK-1	0
IKK	Non-active IKK	1
IKKa	Active IKK	0
NFkB	Free NF-κB	0
mIkBa	IκBα mRNA	0
IkBa	IκBα protein	1
mIL8	IL-8 mRNA	0
IL8	IL-8 protein	0 ng/mL
$IRAK\text{-}1_{tot}$	Total amount of IRAK-1	1
flagellin	Flagellar protein	7.97 or 79.7#
IL1β	IL-1β	1&

S3 Table. Model variables and their initial conditions. *As we measured the relative changes for most molecular species, their concentration units are arbitrary (a.u.) except for IL-8 which concentration unit is ng/mL. We assumed that the whole system was in quiescent condition before it responded to the stimulus, so the initial values of the variables of stimuli-responding molecules were set to zero. For all other variables, we set their initial values to one. # The concentration of flagellin was calculated based on the MOIs of *L. pneumophila* (MOI 10: 7.97 ng/mL and MOI 100: 79.7 ng/mL) used to infect alveolar epithelial cells (the calculation can be found in the supplementary material). & The experimental concentration of IL1-β (1 ng/mL) was used to characterise the corresponding model variable value. When the cells were challenged with only one stimulus, the other one was set to zero.

Parameter	Description	Value[#]	Remark
k_{IRAK1}^{syn}	Synthesis rate of IRAK-1	0.0961	Fixed
k_{IRAK1}^{deg}	Degradation rate of IRAK-1	0.0961	IRAK-1 half-life=~7 h (*54*) [0.0891 0.109]
k_{IRAK1}^{ph1}	Flagellin-mediated phosphorylation rate of IRAK-1	$9.51*10^{-4}$	Estimated[&]
k_{IRAK1}^{ph2}	IL-1β-mediated phosphorylation rate of IRAK-1	3.148	Estimated
k_{IRAK1p}^{deg}	Degradation rate of phosphorylated IRAK-1	7.386	Phosphorylated IRAK-1 half-life= ~6 min (*65*) [6.238 7.624]
k_{IKK}^{syn}	Synthesis rate of IKK	0.146	Fixed
k_{IKK}^{deg}	Degradation rate of IKK	0.150	Estimated
k_{IKK}^{act}	Activation rate of IKK mediated by phosphorylated IRAK-1	83.992	Estimated
k_{IKKa}^{deg}	Degradation rate of active IKK	0.782	Estimated
k_{NFkB}^{gain}	Gain rate of free NF-κB in the nucleus mediated by IκBα and IKKa	0.0120	Estimated
k_{NFkB}^{loss}	The loss of free NF-κB mediated by IκBα	1.717	Estimated
$k_{mIkB\alpha}^{transc}$	NF-κB-mediated transcription rate of IκBα mRNA	540.260	Estimated
$k_{mIkB\alpha}^{deg}$	Degradation rate of IκBα mRNA	1.560	Estimated
$k_{IkB\alpha}^{transl}$	Translational rate of IκBα from IκBα mRNA	19.810	Estimated
$k_{IkB\alpha}^{loss}$	Loss of free IκBα mediated by NF-κB and IKKa	123.757	Estimated
$k_{mIL8}^{transc1}$	Basal transcription rate of IL-8 mRNA	$7.03*10^{-4}$	Estimated
$k_{mIL8}^{transc2}$	NF-κB-mediated transcription rate of IL-8 mRNA	252.633	Estimated
k_{mIL8}^{deg}	Degradation rate of IL-8 mRNA	0.559	Measured *IL-8* mRNA half-life=~82 min [0.457 0. 615]
k_{IL8}^{transl}	Translation rate of IL-8 from its mRNA	0.0748 ($ng*mL^{-1}*h^{-1}$)	Estimated
k_{IL8}^{deg}	Degradation rate of IL-8	0.173[$] ($ng*mL^{-1}*h^{-1}$)	IL-8 half-life=~4 h (*65*)
K_1, K_2, K_3	Michaelis-Menten coefficient	1	Fixed
N_{tot}	Total amount of free NF-κB	1	Fixed

S4 Table. Model parameters and their values. [#] The time scale of the kinetic rate constants is h. [&] the searching interval for the estimated parameters is [$4*10^{-4}$ $2.5*10^{3}$] unless it is specified, e.g. the intervals for k_{IRAK1}^{deg}, k_{IRAK1p}^{deg} and k_{mIL8}^{deg} is 10% variance of their original values obtained from literature. [$] k_{IL8}^{deg} is set to zero during parameter estimation, IL-8 was measured in supernatants, where the absence of cellular proteins like proteases minimized its degradation.

References

1 Berg, J. *et al.* Tyk2 as a target for immune regulation in human viral/bacterial pneumonia. *Eur Respir J* **50**, doi:10.1183/13993003.01953-2016 (2017).

2 Du Bois, I. *et al.* Genome-wide chromatin profiling of Legionella pneumophila-infected human macrophages reveals activation of the pro-bacterial host factor TNFAIP2. *The Journal of infectious diseases*, doi:10.1093/infdis/jiw171 (2016).

3 Heuner, K. & Albert-Weissenberger, C. in *Legionella: Molecular Microbiology* (eds K. Heuner & M. Swanson) Ch. 6, (Caister Academic Press, 2008).

4 Schmidt, H. & Jirstrand, M. Systems Biology Toolbox for MATLAB: a computational platform for research in systems biology. *Bioinformatics* **22**, 514-515, doi:10.1093/bioinformatics/bti799 (2006).

5 Butcher, E. C., Berg, E. L. & Kunkel, E. J. Systems biology in drug discovery. *Nat Biotechnol* **22**, 1253-1259, doi:10.1038/nbt1017 (2004).

6 Krishna, S., Jensen, M. H. & Sneppen, K. Minimal model of spiky oscillations in NF-kappaB signaling. *Proc Natl Acad Sci U S A* **103**, 10840-10845, doi:10.1073/pnas.0604085103 (2006).

7 Raue, A. *et al.* Structural and practical identifiability analysis of partially observed dynamical models by exploiting the profile likelihood. *Bioinformatics* **25**, 1923-1929, doi:10.1093/bioinformatics/btp358 (2009).

8 Jensen, L. E. & Whitehead, A. S. IRAK1b, a novel alternative splice variant of interleukin-1 receptor-associated kinase (IRAK), mediates interleukin-1 signaling and has prolonged stability. *J Biol Chem* **276**, 29037-29044, doi:10.1074/jbc.M103815200 (2001).
